# The protein translocation systems in plants – composition and variability on the example of *Solanum lycopersicum*

**DOI:** 10.1186/1471-2164-14-189

**Published:** 2013-03-18

**Authors:** Puneet Paul, Stefan Simm, Andreas Blaumeiser, Klaus-Dieter Scharf, Sotirios Fragkostefanakis, Oliver Mirus, Enrico Schleiff

**Affiliations:** 1 Department of Biosciences, Molecular Cell Biology of Plants, Goethe University, Max von Laue Str. 9, Frankfurt/Main, 60438, Germany; 2 Cluster of Excellence Frankfurt, Goethe University, Max von Laue Str. 9, Frankfurt/Main, 60438, Germany; 3 Center of Membrane Proteomics, Goethe University, Max von Laue Str. 9, Frankfurt/Main, 60438, Germany

**Keywords:** Translocation machineries, Orthologue search, Plants, Chloroplast, Mitochondria, Peroxisomes, ER, ERAD

## Abstract

**Background:**

Protein translocation across membranes is a central process in all cells. In the past decades the molecular composition of the translocation systems in the membranes of the endoplasmic reticulum, peroxisomes, mitochondria and chloroplasts have been established based on the analysis of model organisms. Today, these results have to be transferred to other plant species. We bioinformatically determined the inventory of putative translocation factors in tomato (*Solanum lycopersicum*) by orthologue search and domain architecture analyses. In addition, we investigated the diversity of such systems by comparing our findings to the model organisms *Saccharomyces cerevisiae, Arabidopsis thaliana* and 12 other plant species.

**Results:**

The literature search end up in a total of 130 translocation components in yeast and *A. thaliana*, which are either experimentally confirmed or homologous to experimentally confirmed factors. From our bioinformatic analysis (PGAP and OrthoMCL), we identified (co-)orthologues in plants, which in combination yielded 148 and 143 orthologues in *A. thaliana* and *S. lycopersicum*, respectively. Interestingly, we traced 82% overlap in findings from both approaches though we did not find any orthologues for 27% of the factors by either procedure. In turn, 29% of the factors displayed the presence of more than one (co-)orthologue in tomato. Moreover, our analysis revealed that the genomic composition of the translocation machineries in the bryophyte *Physcomitrella patens* resemble more to higher plants than to single celled green algae. The monocots (*Z. mays and O. sativa*) follow more or less a similar conservation pattern for encoding the translocon components. In contrast, a diverse pattern was observed in different eudicots.

**Conclusions:**

The orthologue search shows in most cases a clear conservation of components of the translocation pathways/machineries. Only the Get-dependent integration of tail-anchored proteins seems to be distinct. Further, the complexity of the translocation pathway in terms of existing orthologues seems to vary among plant species. This might be the consequence of palaeoploidisation during evolution in plants; lineage specific whole genome duplications in *Arabidopsis thaliana* and triplications in *Solanum lycopersicum.*

## Background

In eukaryotic cells most of the proteins are cytosolically translated and many have to be translocated across at least one membrane to reach their place of action [1,2]. Albeit the mode of translocation differs between different translocation systems, they all have in common that they are composed of multiple subunits. Translocation across the membrane of the endoplasmic reticulum (ER) marks a central event as majority of the proteins delivered to other compartments of the cell have their entry point at this membrane, while peroxisome, mitochondria and chloroplast possess their own system for transporting specific proteins across their membranes. Translocation of proteins in or across the ER membrane takes place either co- or post-translationally, and an ER-associated degradation machinery (ERAD) controls the quality state of the imported proteins [3,4]. Remarkably, the translocon at the peroxisomal membranes are in parts similar to the ERAD system [5-7].

Mitochondria possess at least five distinct pathways for the transport of cytosolically synthesized proteins, namely (i) the “presequence pathway”, (ii) the “carrier pathway”, (iii) the “intermembrane space assembly (MIA) pathway”, (iv) the “sorting and assembly of outer membrane β-barrel proteins (SAM) pathway”, and (v) the insertion mode for α-helical outer membrane proteins by the mitochondrial import protein 1 [8,9]. All with the exception of pathway (v) unify at the translocase of the outer membrane of mitochondria (TOM complex).

Similar to mitochondria, chloroplasts import several hundreds of nucleus-encoded proteins [10]. The preproteins are recognized by the TOC complex, delivered into the intermembrane space (IMS) and further transported by the TIC complex [9]. Recently, a Sec system was identified in the inner chloroplast envelope [11]. Within chloroplasts, proteins are further targeted to the thylakoid membranes or lumen.

Thus, protein translocation into or across membranes requires the action of many different proteins. At stage, a general inventory of these proteins in plants has not been established; only few cases have been presented mostly for the model plant *A. thaliana*[12,13]. Thus, we aimed at analyzing the complexity and conservation of protein translocation systems in different plant species that have not yet been biochemically approached. For this purpose we performed a bioinformatic BLAST-dependent orthologue search for putatively involved candidates in 13 other plant species on base of the model systems *A. thaliana* and yeast. We obtained an inventory of orthologues and (co-)orthologue. The findings were validated by analyzing the domain architecture, the subsistence of shared synteny and expression in different tissues of Arabidopsis and tomato, which facilitates the correlation of orthologues and the search for functional connotation. In this study, we concentrated on the relationship of genes due to lineage-specific duplication(s) and skipped the time-point of the duplication in relation to speciation, which is necessary to call them paralogues [14]. So, we concentrate only at the genomic level and thereby used the terms orthologues and (co-)orthologues in our analyses.

The results are exemplified in more detail for tomato, an economically important crop and the model plant for studying fleshy fruit development and ripening as well as wound response [15-17]. We give an overview concerning conservation and diversification in translocation machineries of different cellular compartments in a cell during genome evolution of Viridiplantae. Further, we highlight inter-species differences in the conservation of translocons in plants and between plant and yeast in general. We demonstrate that among all the compartments the chloroplast translocases are most conserved with one to one relations in the orthologues from *A. thaliana* to *S. lycopersicum*.

## Results and discussion

### The database structure and factor analysis

To gain insight into the molecular composition of the protein translocation systems in intracellular membranes of plant cells, we generated a bioinformatic pipeline for the identification of orthologues and (co-)orthologues in plants. Primarily, a manual data-mining process was established to collect central components involved in protein translocation based on existing literature in Arabidopsis and yeast (step i). The identified components were used to extract their amino acid sequences (step ii). Then, BLAST dependent orthologue search was used to identify (co-)orthologues in other plant lineages (step iii) and to assign the different (co-)orthologues of specific factors via their domain structure, synteny and expression pattern (step iv).

We identified a total of 130 translocation components in yeast and *A. thaliana* from our literature-based search (Additional file 1). The two approaches (PGAP and OrthoMCL) were utilized to identify (co-)orthologues in plants, which in combination yielded 148 and 143 orthologues in *A. thaliana* and *S. lycopersicum*, respectively (Additional files 2, 3 and 4). Interestingly, we traced 82% overlap in findings from both approaches though we did not find any orthologues for 27% of the factors by either procedure. The latter was majorly accounted by factors of the ER system (11 factors). In turn, 29% of the factors displayed the presence of more than one (co-)orthologue in tomato.

**Figure 1:**
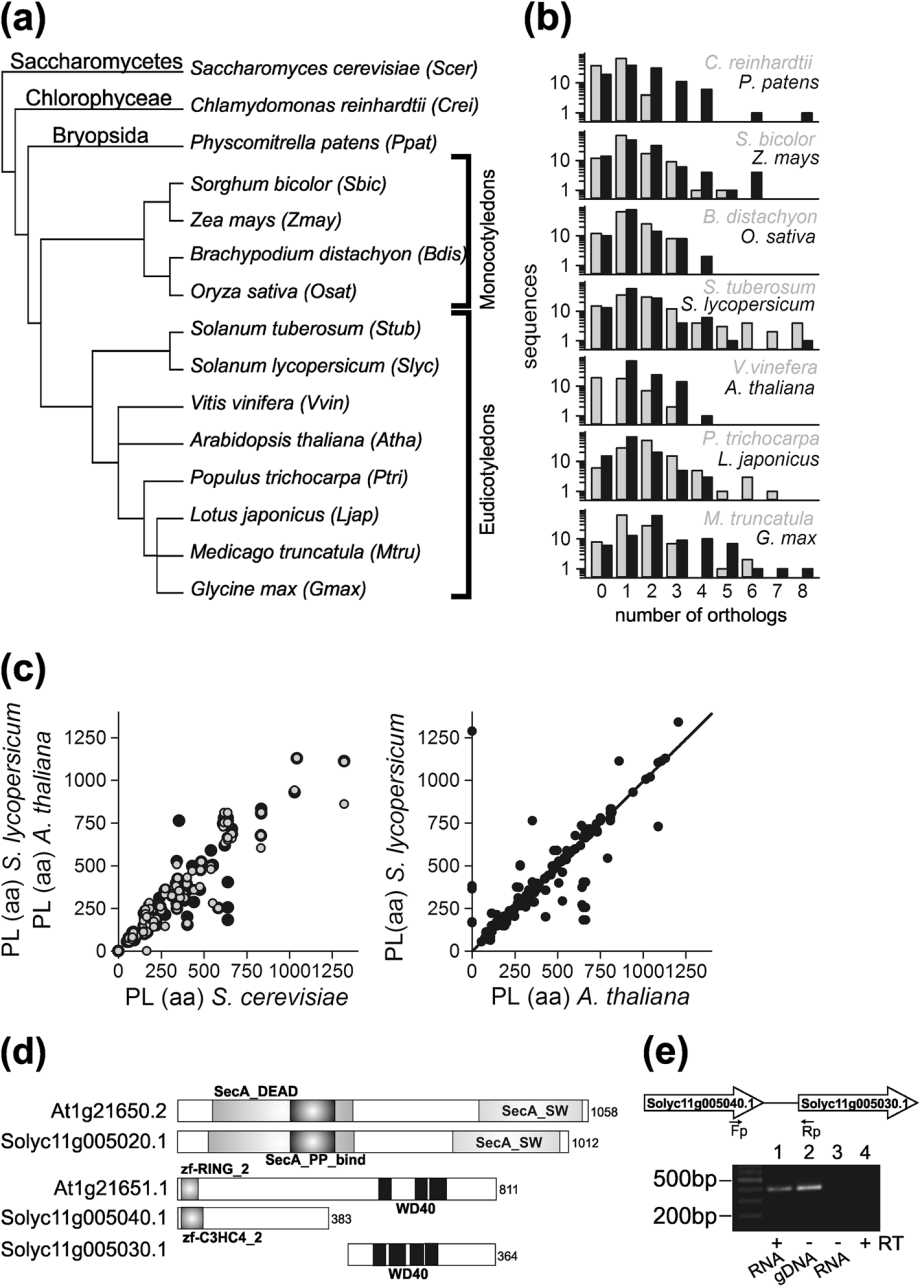
**The analysis of the orthologous species.** (**a**) The phylogenetic relation of the plant species analysed via OrthoMCL (Additional files 2 and 4) is given. (**b**) Correlation of the number of protein sequences to the number of orthologues for all 14 plant species discussed (**c**) The according orthologues in *S. lycopersicum, S. cerevisiae* and *A. thaliana* (left scatterplot) and *S. lycopersicum* to *A. thaliana* (right scatterplot) have been analysed with respect to amino acid number (protein length in amino acids). The line in the right scatterplot represent the least square fit analysis to y=a*x with a=0.992. (**d**) Domain architecture of cpSecA2 orthologues from *A.thaliana* and *S. lycopersicum*. AT1G21650 corresponds to Solyc11g005020 whereas AT1G21651 corresponds to the combination of Solyc11g005040 and Solyc11g005030. (**e**) RT-PCR results confirming that Solyc11g005040 and Solyc11g005030 is ‘one gene’; lanes 1 to 4: cDNA, genomic DNA, no-RT control (without reverse transcriptase), negative control (water), respectively.

With respect to the other plant species (Figure 1a and Additional files 2 and 4) we realized only slight differences in the overall architecture of protein translocation machineries in the respective sub-groups (lower plants, monocots and eudicots). Remarkably, in *Chlamydomonas reinhardtii* most factors are encoded by a single gene with the exception of Erv1 (2 genes), Pex12 (2), cpSecA (2) and Tic20 (3). Moreover, *Physcomitrella patens* seems to have a more similar composition of translocation machinery components to higher plants in contrast to the single celled green algae *Chlamydomonas reinhardtii*. The monocots follow more or less a similar conservation pattern than the eudicots for encoding the translocon components, although few distinctions were observed concerning the number of (co-)orthologues (*Z. mays, O. sativa*). In contrast, a diverse pattern was observed for the eudicots. Generally, we observed higher numbers of (co-)orthologues in *Glycine max* (eudicot) and *Z. mays* (monocot)*,* which may be related to their larger genomes.

We also realized that certain factors could not be identified in individual species, namely Sec65 (*Lotus japonicus*), Hrd3, Dfm1, Pex22 and Pex1 (*Solanum tuberosum*) and others (Additional files 2 and 4), which could be due to limitation of draft genomes for the respective plants. Remarkably, Pex14 could not be identified in 11 of the 14 analyzed genomes, while Pex13 is not found in *C. reinhardtii* and *P. patens*. In addition, we observed that some components were encoded by an increasing number of genes across the groups and species, especially Toc159, Toc75, Tic20, Srp54, Ubc5 and Cdc48. We also correlated the number of (co-)orthologues found in 14 plant species to the number of protein sequences for the translocation factors (Figure 1b), which give hints for whole genome duplications and the conservation of the translocation factors in general.

It is evident that certain factors are represented by multiple orthologues (e.g. chloroplast translocation machinery in *A. thaliana*). To explore the relationship between the different factors, we studied the sequences in more detail. At first, we determined the protein length based on the identified amino acid sequence (Additional files 5, 6, 7, 8 and 9), because the Tomato Genome Consortium (2012) reported a similar amino acid sequence length for ~43% of tomato proteins with their respective Arabidopsis orthologues. In line with this notion, we found a good correlation pattern between amino acid length of the translocon components from tomato and Arabidopsis (Figure 1c).

Further, we probed for predictable functional domains of the identified components (Additional files 5, 6, 7, 8 and 9). In cases where more than one domain was predicted, the order of the domains was automatically analyzed. For 77% of all proteins at least once the same domain architecture for an Arabidopsis and a tomato protein in the same group of orthologues was observed. However, the domain architecture enabled us to assign one cpSecA2 orthologue in Arabidopsis (AT1G21650) to Solyc11g005020 whereas the other orthologue AT1G21651 (zinc finger-RING_2_domain; 3x WD40 domain) could be similar to the combination of Solyc11g005040 (zinc finger-C3HC4_2 domain) and Solyc11g005030 (3x WD40 domains; Figure 1d). Thus, to prove the latter, we designed the forward primer from exonic region (3^′^-end) of Solyc11g005040 and the reverse primer from exonic region (5^′^-end) of Solyc11g00530. We observed amplicon of ~400bp by RT-PCR on isolated RNA (Figure 1e, lane 1) and by PCR on genomic DNA (lane 2). In the absence of the reverse transcriptase (lane 3) or RNA (lane 4) no product is observed. Thus, our results suggest that the two genes annotated represent a single gene (Additional file 10).

Nevertheless, after the two steps we still had 38 cases, where we could not clearly assign the orthologues from Arabidopsis and orthologues from tomato to each other. Thus, we applied additional strategies. First, we inspected publically available expression data to assign pairs of genes that are similarly regulated in both *A. thaliana* and tomato (Additional files 11, 12, 13 and 14). However, the success was rather limited as the data density for tomato is not comparable to the one for *A. thaliana*. Secondly, we compared the genomic region of the bait and the identified orthologue. For this, we analyzed the shared synteny for a region of seven genes up and downstream of the particular factor in Arabidopsis and tomato (Additional file 15). This approach assisted in the assignment of the tomato (co-)orthologues to specific Arabidopsis genes. Although for many of the factors the syntenic score appeared to be zero, some gene pairs that could be clearly assigned as orthologues via this analysis are indicated in (Additional files 5, 6, 7, 8 and 9).

### The co-translational translocation system at the ER membrane

In this pathway, the ribonucleoprotein complex annotated as signal recognition particle (SRP) binds to the hydrophobic region of the targeting signal of the emerging polypeptide. SRP mediates targeting of the ribosome-nascent chain complex (RNC) to the membrane bound SRP receptor (SR) [18] composed of SRα and the integral membrane protein SRβ [19]. The RNC is then transferred to the channel Sec61 [20]. The transfer of the RNC to the Sec61 complex is coupled with dissociation of SRP-SR complex making the components available for a new round of protein translocation [3].

**Figure 2:**
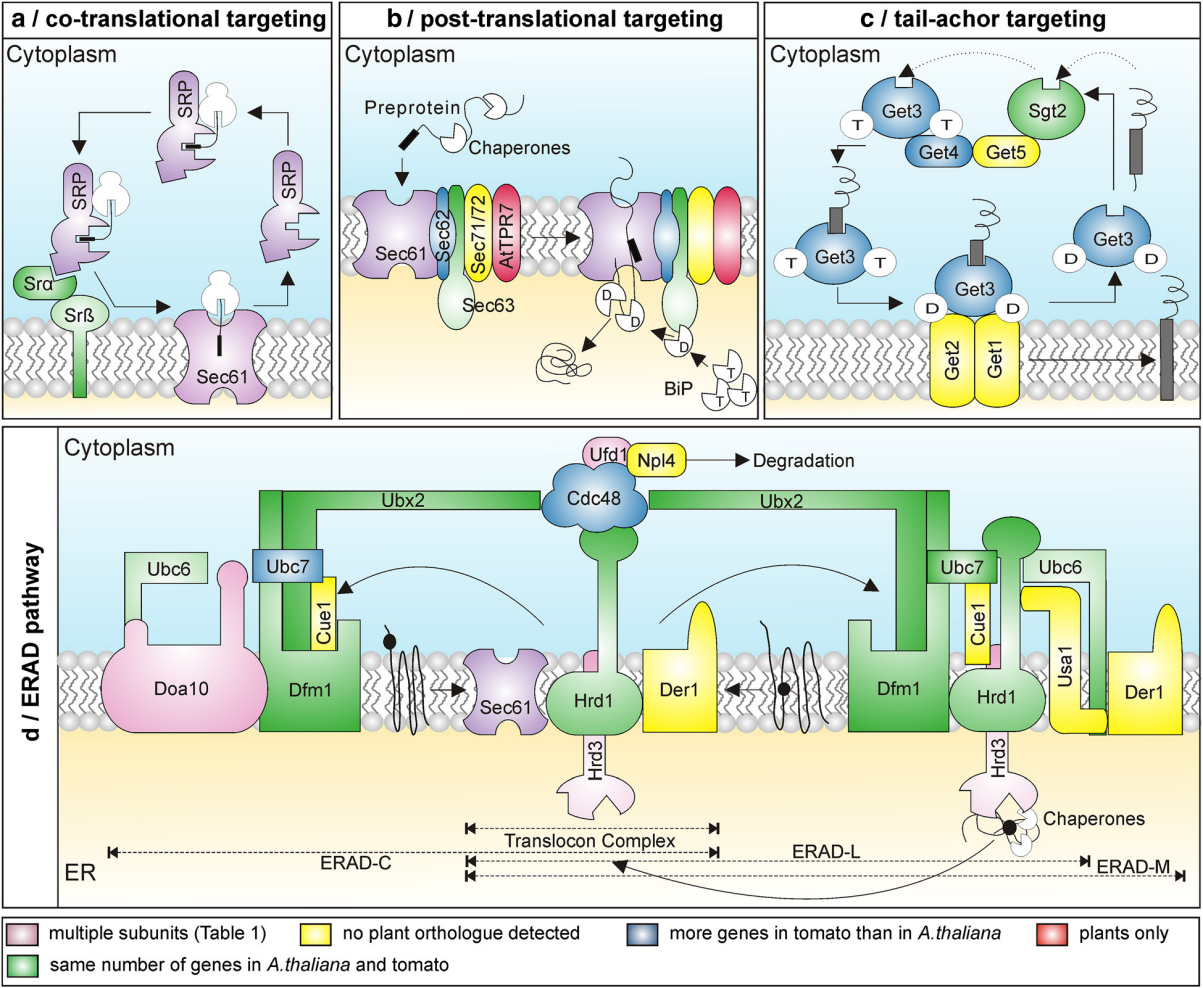
**The ER & ERAD translocation system according to yeast.** (**a**) In the co-translational pathway, SRP binds to the emerging polypeptide to form a RNC. Then, SRP is recognized by the SR composed of SRα and SRβ. The RNC is transferred to Sec61, which translocates the emerging protein into or across the ER membrane. (**b**) In the post-translational pathway, the precursor is guided by chaperones to the Sec62/63 and Sec61 complex. The final insertion or translocation of polypeptides is assisted by BiP, the ER-resident Hsp70 isoform [21]. (**c**) TA proteins are transferred to Get3 in a Sgt2/Get4/Get5 mediated manner. The Get3-TA protein complex interacts with Get1-Get2 complex, the latter facilitating the release of TA protein into the membrane. (**d**) In the ERAD-L pathway, misfolded substrates are recognized by ER-resident chaperones. This complex interacts with the luminal domain of Hrd3, the latter being complexed with the Hrd1/Der3 [22]. Their substrates are translocated via Sec61, Der1 or the E3 ligases. A complex of cytosolic E3-ligases (anchored to the membrane; Doa10, Hrd1/Der3) and E2-conjugating enzymes (Ubc6, Ubc7 bound to Cue1) ubiquitinate the substrates in the cytosol. Hrd1/Der3 interacts with Der1 via Usa1, while Dfm1 forms complexes with Doa10, Hrd1/Der3 and Cdc48. After ubiquitination, the substrates are pulled out by the AAA ATPase machinery (Cdc48, in complex with Npl4, Ufd1 and Ubx2) and degraded by the 26S proteasome. Note: The colour indicates the number of genes encoding the component. Green: equal number of genes found in Arabidopsis and tomato; blue: more genes in tomato than in *A. thaliana*; pink: less genes in tomato than in *A. thaliana*; yellow: no orthologues found in plants; red: only found in plants; purple: multiple subunits unified for better presentation; brown: not found in tomato; white: not included in our discussion.

Multiple (co-)orthologues are found for the SRP components Srp72 and Srp54 in plants (2/4 and 3/2 in *A. thaliana* and tomato, respectively; Figure 2a, Additional file 5). While the putative Srp54 proteins from tomato possess a comparable length and domain architecture to their Arabidopsis counterparts, only one identified tomato Srp72 (Solyc11g062270) is comparable to the *A. thaliana* protein, while the tomato Srp72 (co-)orthologue (Solyc01g047590) does not contain the typical “SRP72 DOMAIN”. For Sec65p a single orthologue is detectable in tomato, which is longer than the *A. thaliana* orthologue, but both proteins are shorter than the corresponding protein in yeast. The receptor complex ‘SRα/SRβ’ is found in all plants as well.

For all other SRP constituents, we did not find orthologues in any analyzed plant. However, by BLAST search analysis two genes similar to Srp14p were identified in *A. thaliana* (AT3G49100, AT2G43640) and one in tomato (Solyc12g099820). Closer inspection revealed that AT3G49100 and Solyc12g099820 contain the “SRP9-21 DOMAIN” and are orthologous to each other, while AT2G43640 contain the “SRP14 DOMAIN”. Thus, it might be speculated that one gene exists each for Srp21 and Srp14 in Arabidopsis*,* while the tomato genome only encodes for Srp21.

### The post-translational translocation system at the ER membrane

In the post-translational pathway, preproteins are guided by chaperones to Sec61 via a complex composed of Sec62p, Sec63p, Sec71p and Sec72p. We identified orthologues for Sec62 and Sec63 in all plants analyzed (Figure 2*,* Additional file 5). One orthologue in *A. thaliana* and two in tomato are identified for Sec62, while Sec63 has two orthologues in both plant species. As previously reported [23], we could not identify orthologues for Sec71 and Sec72, which interact with chaperones via tetratricopeptide repeat (TPR) domains. However, Sec72 might be replaced by the TPR containing ER-protein atTPR7 (AT5G21990; Solyc06g073840), which interacts with Sec63 in *A. thaliana*[24].

The Sec61 translocon in yeast is composed of Sec61p, Sbh1p and Sss1p, or alternatively of Ssh1p, Sbh2p and Sss1p [3]. In *A. thaliana* we found three orthologues to all components except of Ssh1p (Additional file 5). In tomato we detected only two orthologues for Sec61p and Sbh1p/Sbh2p, and none for Sss1p. On the contrary, orthologues to Sss1p were found in all other plant species. Thus, we decided to use simple BLAST via which we identified a putative Sss1p (Solyc05g050720) in tomato. The protein contains the characteristic “SecE DOMAIN”, while its sequence is about three times as long as the *A. thaliana* equivalents (AT5G50460, AT4G24920, AT3G48570). Further, we realized that Sbh1p is absent from all the monocots except rice while Sbh2p is encoded by the genomes of all the monocots suggesting redundancy in Sec61 subunits (Additional file 2). Interestingly, from the ‘shared synteny’ analysis we could relate two (co-)orthologues of Sec61 from tomato and Arabidopsis together (AT1G78720/Solyc10g007390, AT1G29310/Solyc02g072130), whereas the third Arabidopsis orthologue (AT2G34250) may have originated from a recent duplication.

### The translocation of tail-anchored proteins at the ER membrane

Proteins with single C-terminal transmembrane domain (TMD; tail-anchored (TA) proteins) are recognized by yeast Sgt2 and transferred to the cytosolic Get3 in a Get4-Get5-mediated manner (Figure 2c) [25]. The two membrane proteins Get1 and Get2 interact with the Get3-TA protein complex and thereby mediate the TA protein release into the ER membrane [26,27].

In plants, we identified Sgt2, Get3 and Get4. In *A. thaliana* we found in general one orthologue, whereas we observed one orthologue of Sgt2, two of Get4 and four of Get3 in tomato. However, it has to be mentioned that one of the Get3 orthologues in tomato (Solyc05g050490) is significantly longer than the protein in *A. thaliana* and yeast and possess an additional “ANION TRANSPORTING ATPase DOMAIN” beside the “Pkinase DOMAIN”. Moreover, from the shared syntenic analysis, we could correlate one of the orthologue of Get3 from tomato (Solyc01g091880) to the Arabidopsis orthologue (AT1G01910). Interestingly, Solanaceae family members were found to possess more genes for Get3 than other plant species (*S. tuberosum,* 6*; S. lycopersicum,* 4) Additional file 1.

### The ER-associated degradation machinery

Proteins having misfolded cytosolic, luminal or membrane-spanning domains are exported either by ERAD-C, ERAD-L and ERAD-M pathways, respectively (Figure 2d, Additional file 6) [4,28]. ERAD-L substrates are first recognized by ER-resident chaperones and subsequently translocated to the cytosolic face of the ER membrane. For this process, Sec61 [29], Derlins [30] and the E3 ligases [31] have been proposed as possible dislocases. During transfer, membrane bound RING-finger E3-ligases and E2-conjugating enzymes install ubiquitin moieties on the substrates. Once ubiquitylated, these misfolded substrates are pulled by an AAA ATPase complex (Cdc48, Npl4, Ufd1, Ubx2), which further hands them over to the 26S proteasome for degradation [32].

For the ERAD-C, we identified tomato orthologues to the E3 ligase Doa10 (1) [33], the E2-conjugating enzymes Ubc7 (4) and Ubc6 (2), but not to Cue1, which is thought to recruit Ubc7 to the ER membrane in yeast [34]. From the shared syntenic analysis, we could correlate orthologues of Doa10 in *Arabidopsis* and tomato (AT4G34100/Solyc01g107880).

The described components of the ERAD-L and ERAD-M system are all present in plants with the exception of Usa1, which in yeast promotes the interaction between Hrd1/Der3 and Der1 [35]. However, Npl4 and Ubx2 in Arabidopsis have only been detected by literature search, but not by the defined pipeline. Orthologues to Npl4 from Arabidopsis (AT2G47970, AT3G63000) are detectable in the genome of tomato (Solyc06g084440), but not between Npl4 (YBR170C) in yeast to other plant species. Furthermore, we did not detect orthologues to Ubx2 (YML013W) in Arabidopsis, although a functionally related protein (PUX1: AT3G27310) was previously described [36]. No orthologue in *Arabidopsis* was found for Der1, thus it might be that Dfm1 [30] and Der1 function is performed by a single protein in plants, while in yeast Der1 acts in the ERAD-L and ERAD-M path and its homologue Dfm1 (At4g29330; Solyc05g026310) in the ERAD-C path.

For Hrd1/Der3 [37,38] that acts as E3 ligase in the ERAD-L and ERAD-M path, we identified two orthologues for 8 of the 14 plants (including *A.thaliana* and *S. lycopersicum*), while the others possess either one or three (co-)orthologues. However, one of the *A. thaliana* proteins (AT1G65040) is significantly shorter than the other proteins. We noticed that this Hrd1 from *A. thaliana* showcases three splice variants, which differ in their amino acid lengths to a large extent (AT1G65040 i1: 281 aa; i2: 480 aa; i3: 389 aa). Thus, the longest relates to the orthologues in yeast and tomato. For Hdr3 and Ufd1 we identified two/three orthologues in *A. thaliana*, but only one in tomato. From the syntenic scores, we suggest the two Ufd1 orthologues (AT2G21270 and AT4G38930) from Arabidopsis corresponds to one gene in tomato (Solyc01g110410). Interestingly, plant species possess higher number of Cdc48 genes (as high as 10 in *G. max*), which may be accounted for its role in multiple processes (Additional file 2).

### The translocation system at the peroxisomal membrane

Peroxisomal proteins are translocated across or inserted into the membrane by three distinct pathways [39]. (i) Some peroxisomal membrane proteins (type-I PMPs; e.g. Pex3 and Pex15) with an N-terminal membrane-targeting signal (mPTS) lacking the Pex19-binding site [40]. (ii) Type-II PMPs (e.g. PMP22) with Pex19-binding motif–containing mPTS [40,41] are recognized by the cytosolic Pex19, which docks to Pex3 while releasing the bound PMP into the membrane [39]. (iii) Peroxisomal matrix proteins contain either a C-terminal (PTS1) or N-terminal (PTS2) targeting signal [42,43]. The PTS1 is recognized by the soluble receptor Pex5 [44], while Pex7 is the receptor for the PTS2 signal [45]. The receptor-cargo complex is recognized at the peroxisomal membrane and is transferred into the peroxisomes through a ‘transient pore’ formed by Pex5 together with its docking partner Pex14 [46], while the mechanism for PTS2 cargos is yet to be deciphered. The re-export of Pex5 into the cytosol requires ubiquitination by the AAA ATPases Pex1 and Pex6 [47,48].

**Figure 3:**
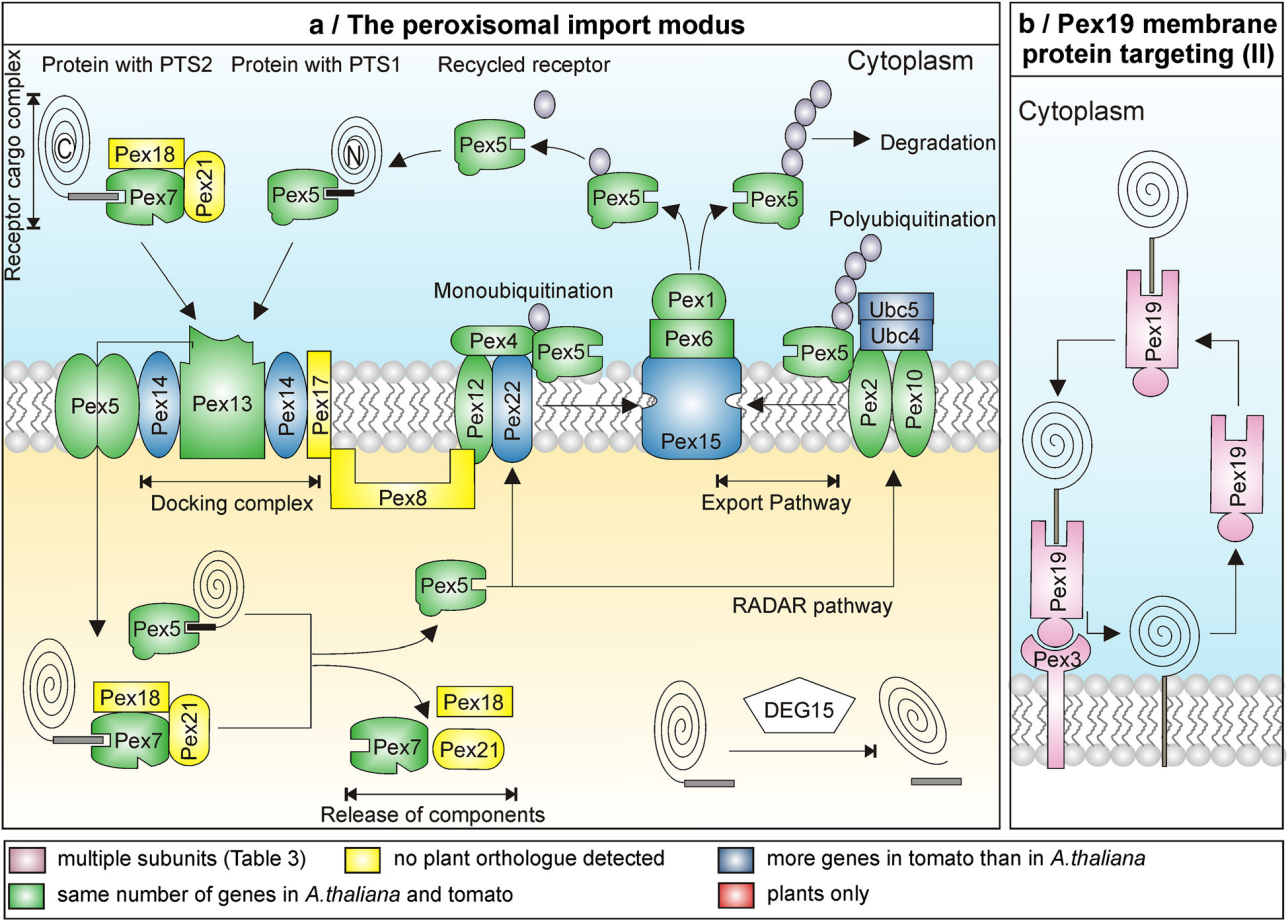
**Peroxisomal protein import.** Pex5 and Pex7 recognize proteins with PTS1 and PTS2, respectively. Pex18 and Pex21 act as co-receptors for Pex7. The receptor-cargo complex is recognized by Pex13, Pex14 and Pex17. Pex5 shuttles between cytosol and membrane (and may be lumen, which at state is controversially discussed), and mediates the transfer of the cargo protein and becomes subsequently monoubiquitinated by Pex22-anchored Pex12 and Pex4. Pex5 is exported by Pex1 and Pex6 (anchored by Pex15). Malfunction of Pex5 recycling induces the RADAR mechanism, by which Pex5 becomes polyubiqunated via the E2-conjugating enzyme Ubc4 or Ubc5 and the E3-ubiquitin ligase Pex2 and Pex10. The polyubiquitinylated Pex5 is exported and degraded in the cytosol. (**b**) Type-II PMPs interact with Pex19, which docks to Pex3 and releases the PMP into the membrane. For color code see legend of Figure 2.

We identified one orthologue each for the central receptors Pex5 and Pex7 in *A.thaliana* and tomato (Figure 3, Additional file 7), while the two Pex7 associated factors Pex18p and Pex21p described in yeast [49] could not be detected. Moreover, Pex5 was missing from the *G.max* genome whereas Pex7 was absent from *V. vinifera*. The membrane bound docking complex composed of Pex13, Pex14 and Pex17 in yeast [50] has also been isolated in *A.thaliana* (atPex13, atPex14) [51]. However, its components cannot be considered as orthologous to those from yeast (Additional file 2). Based on the *A. thaliana* sequences we identified one orthologue for Pex13 and two orthologues of Pex14 in tomato. One of the two Pex14 (co-)orthologues in tomato (Solyc03g114330) is 128 amino acids shorter than the other (co-)orthologue (Solyc06g071470) but possess the same predicted “Pex14 DOMAIN”. However, in Solyc03g114330 this domain is 20 amino acids shorter. Interestingly, Pex14 was absent from all the monocots considered in our study, which questions its central role as part of a potential translocation channel for PTS1 containing proteins, a function which was proposed for the protein from *Leishmania donovani*[52].

All enzymes involved in mono- and polyubiquitination as well as in the export of Pex5 exist in *A. thaliana* and tomato. This suggests that the same translocation mechanism exists in plants and yeast, which involves Pex5 monoubiquitination after cargo delivery as prerequisite for its export to the cytosol. Monoubiquitination is catalyzed by the RING-finger peroxin Pex4 (E2 conjugating enzyme), and the E3 ligase Pex12 [53]. In yeast, Pex22 anchors Pex4 to the peroxisomal membrane [50]. From the defined pipeline, we could not identify Pex22 orthologues, but the protein has previously been identified in *A. thaliana*[54], to which orthologues can be found in all other plant species except *S. tuberosum*.

In parallel, impaired Pex5 recycling leads to its accumulation in the peroxisomes followed by degradation, which was designated as receptor accumulation and degradation (RADAR) pathway [53]. Thereby, Pex5 becomes polyubiquitinated by the E2-conjugating enzyme Ubc4 or Ubc5 and the E3-ubiquitin ligase Pex2 or Pex10 [55], exported via Pex1-Pex6 and targeted to the proteasome, which all exist in plants.

Remarkably, for Pex15 which tethers the AAA ATPases Pex1/Pex6 to the membrane [56] and which thus performs a similar function as Pex22, plant orthologues could not be identified as well, but the protein has also previously been identified in *A. thaliana*[54]. As for Pex22, two Pex15 (co-)orthologues have been recognized in tomato.

The transport of peroxisomal membrane proteins of type II requires the action of Pex3 and Pex19, for which we identified orthologues in plants (2 in *A. thaliana;* 1 in tomato). Furthermore, from the shared synteny analysis, one of the Pex3 orthologues from Arabidopsis (AT3G18160) related more to the only orthologue in tomato (Solyc03g119030). Closer inspection revealed that Pex3 and Pex19 are absent in *C. reinhardtii* and monocots have only one orthologue for Pex3 and Pex19 except *Z. mays*, which possess two for Pex19 (Additional file 3).

### The mitochondrial translocation system

**Figure 4:**
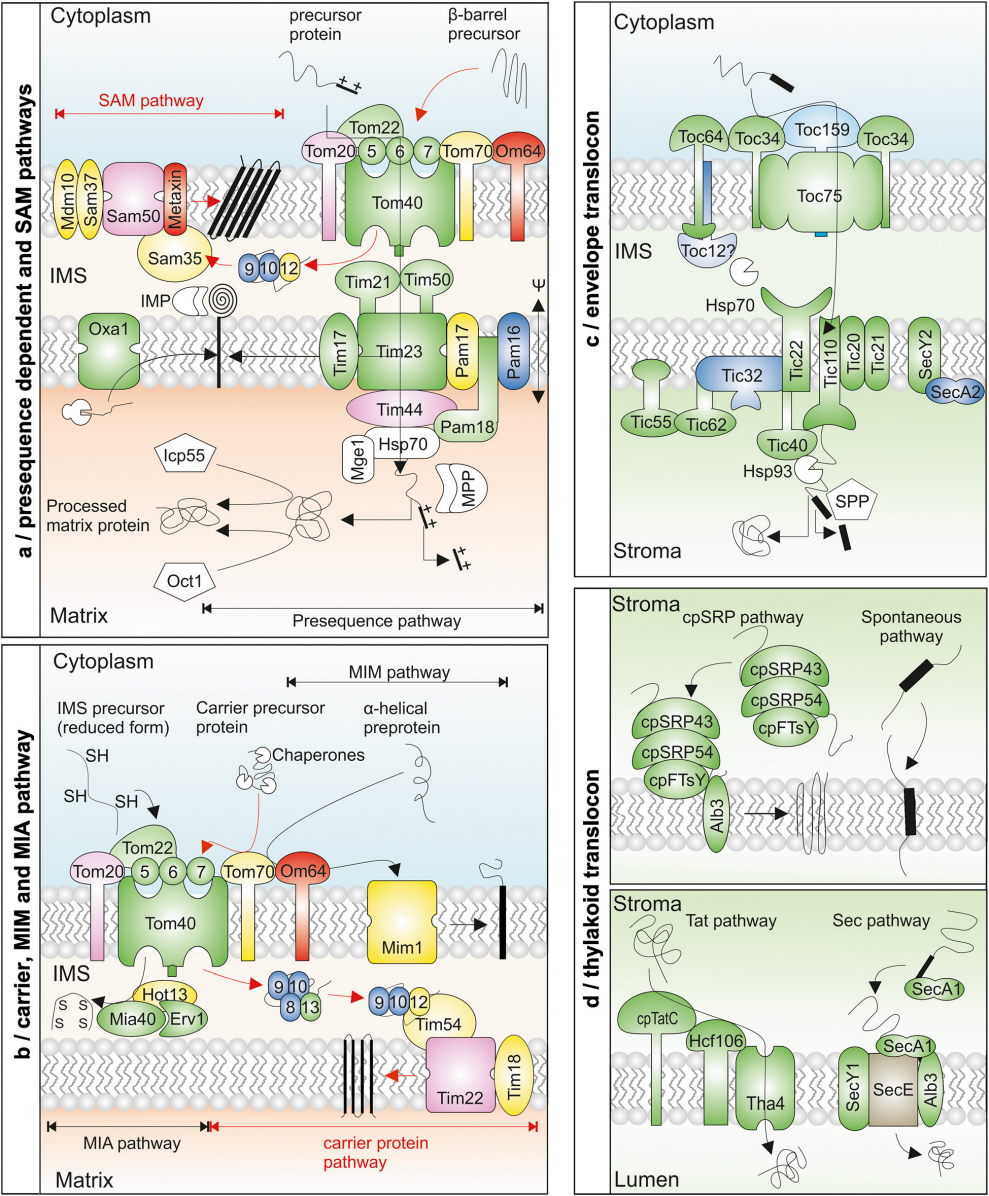
**Mitochondrial and chloroplast protein import machineries.** (**a**,**b**) Import of mitochondrial proteins generally unifies at TOM complex. Tom20 acts as initial receptor for preproteins and transfers them to Tom22 and Tom40 channel. (**a**) Presequence pathway: in IMS preproteins interact with Tim50 / Tim21 and Tim17 / Tim23 translocates preproteins across IM assisted by mtHsp70, Tim44, Pam18, 16 and 17. SAM pathway: in IMS Tim9-Tim10 and Tim8-Tim13 guide β-barrel proteins to SAM complex (Sam50, Sam35, Sam37). In plants, Sam35 and Sam37 are likely replaced by metaxin. (**b**) Carrier pathway: IMPs with cleavable sequence are recognized by OM receptor Tom70 (replaced by OM64 in plants). In IMS Tim9-Tim10 / Tim8-Tim13 guide protein to TIM22 complex, laterally releasing the protein into the membrane. Mia pathway: Mia40 recognizes cysteine residue in IMS preproteins and leads to the formation of a disulfide bond in an Erv1/Hot13-dependent manner. The Mim pathway promotes the insertion of α-helical proteins into the outer membrane. (**c**) Preproteins bind to Toc64, Toc159 and Toc34, which transfer them to Toc75. Toc64 forms ‘IMS complex’ with Toc12, imsHsp70 and Tic22. Tic110, Tic20 and Tic21 have a discussed translocation-channel function, while Tic62, Tic55 and Tic32 regulate the translocon activity. Tic40, stHsp70 and stHsp93 provide energy for final translocation. (**d**) cpSRP pathway: cpSRP is composed of cpSRP54 and cpSRP43, which form a transit complex with substrates and targets them to thylakoid membrane via cpFtsY and Alb3. Spontaneous pathway does not require proteinaceous factors. Twin-arginine translocon: TAT signal containing preproteins bind to Hcf6 and cpTatC, leading to the assembly with oligomeric subunit, Tha4 and transient translocon formation. Sec pathway: cpSecA binds to signal sequence and guides it to cpSecY, cpSecE and Alb3. For color code see legend of Figure 2. The question mark on Toc12 indicates that localization of this factor is under debate.

Most of the mitochondrial import pathways require the action of the TOM complex localized in the outer membrane, which is composed of Tom20, Tom22, Tom40, Tom5, Tom6 and Tom7. Tom20 acts as an initial receptor for N-terminal presequences [57], which are then transferred to Tom22. The preprotein is translocated through the Tom40 channel [58]. Tom5, Tom6 and Tom7 are thought to modify the assembly and stability of the TOM complex [9]. The general importance of this translocon for the subsequent events might explain that all TOM components are present in plants, considering that Tom70 is replaced by OM64 (Figure 4a and b, Additional file 8) [59]. Tom70 is involved in the recognition of inner membrane proteins delivered by Hsp90 chaperones [60]. On the contrary, PGAP only predicted orthologues for Tom70 in yeast (YNL121C, YHR117W) and Arabidopsis (AT1G62390, AT5G20390), but these proteins have a “PB1 DOMAIN”, which adopts an ‘ubiquitin-like b-grasp structure’ that is involved in protein-protein interactions [61] comparable to the TPR folds typically found in Tom70. Further, AT1G62390 was identified as cytosolic- localized CLUMPED CHLOROPLASTS 1, which is involved in chloroplast biogenesis [62]. Thus, the two identified proteins might have similar evolutionary roots, but do not represent functional equivalents.

Tom20, Tom22, Tom5 and Tom7 were not found by orthologue search, but by literature search [63]. While 4 orthologues of Tom20 have been described in Arabidopsis, we could only detect 2 (co-)orthologues in tomato, while other plants also encode for the same in the range of 1–4 (co-)orthologues. For the other three factors we identified the same number of orthologues in tomato and Arabidopsis. However, we could not detect any orthologue for Tom5, Tom6 and Tom7 in *C.reinhardtii* and *M.truncatula* whereas Tom22, Tom6 and Tom7 were absent from the genome of *V.vinifera*. Tom40 and Tom6 could be identified by the orthologue search and identical numbers (2/1, respectively) were identified in both plant genomes. We also identified the existence of Tom40 in all other plants as well but with varied number of orthologues.

The preprotein transported by the TOM complex and emerging in the intermembrane space (IMS) interacts with the TIM23 complex (translocases of inner membrane of mitochondria), which consists of Tim50, Tim23, Tim21 and Tim17. Tim21 induces presequence-release from Tom22 and Tim50 acts as primary receptor in the IMS [64,65]. Thereafter, the preprotein is translocated across the inner membrane through the Tim23 channel [66] or laterally released from Tim23 by the action of Tim17 [67]. The import motor constituted by the matrix protein Tim44, the presequence translocase-associated motor components (Pam18, Pam16, Pam17), and mtHsp70 drives the translocation across the inner membrane [9].

All components of the TIM23 complex can be identified. For Tim23 and Tim17 we found three and two orthologues in *A. thaliana* and tomato, respectively. Remarkably, the genes identified for Tim23 are in general 21 kDa (~190 amino acids). One orthologue in tomato (Solyc10g024350) has even only 151 amino acids and carries a “Tim17 DOMAIN” that starts 25 amino acids prior to other orthologues, which might hint to a false annotation in the genome of tomato. For Tim50 we identified at least one orthologue in yeast as well as all plant species discussed. While orthologues to yeast Tim21 could not be detected, but [63] previously reported it in Arabidopsis (AT4G00026), with which we found orthologues in all other plant species.

The plant orthologues to the yeast components of the PAM complex have been generally identified with the exception of Pam17. We report the existence of Pam18 and Mdj2 in the same orthology group with three orthologues. For Pam16 and Tim44 we observed distinct numbers of orthologues in the different plant species, but we could not identify Tim44 orthologue for *L. japonicus*.

The TIM22 complex composed of four subunits (Tim54, Tim22, Tim18, Tim12) is involved in the insertion of inner membrane proteins [68]. The preproteins are transferred by the tiny TIM proteins to the receptor Tim54 and the channel-forming Tim22 [9]. The precursor proteins are then laterally released from the Tim22 channel into the inner membrane. While Tim22 could be identified (e.g. 2 in *A. thaliana* / 1 in tomato; Additional file 8), Tim54, Tim18 and Tim12 were not found (Additional file 2). The tiny Tims functioning as IMS chaperone for translocation can be identified in all plants, except the absence of Tim9 from the genome of *L.japonicus*. From the shared synteny analysis, one of the Tim9 orthologue from tomato (Solyc10g086510) showed a clear affiliation to the only orthologue from Arabidopsis.

Inner membrane proteins are also inserted from the matrix side by the action of the Oxa1 proteins. The pathway exists in plants as well, as two orthologues of Oxa1 can be found in both, *A. thaliana* and tomato. However, one of the orthologues in tomato is rather small (201 amino acids; Solyc08g082290) and has a shorter “60KDa inner membrane protein DOMAIN” characterizing the Oxa1 protein family.

Preproteins targeted to the IMS contain conserved hydrophobic clefts and cysteine residues, which are recognized by Mia40 leading to the formation of a disulfide bond in an Erv1 dependent manner [69]. The protein Hot13 maintains Mia40 in the zinc-free state to promote its oxidation by Erv1 [70]. The complex seems to be conserved between plants and yeast, as the two essential components, Mia40 and Erv7 were identified in the genome of almost all plants analyzed here (Additional file 2, 4 and 8). Only in the genome of *C. reinhardtii* we could not find Mia40.

### The insertion of proteins into the outer mitochondrial membrane

Outer membrane β-barrel proteins are translocated into the IMS and guided by small Tim chaperones Tim9-Tim10 and Tim8-Tim13 to the SAM complex [68]. This complex is composed of the Omp85-like translocation pore. Sam50, the receptor Sam35 and the gating factor Sam37 [71]. In addition, Mdm10 was discussed to collaborate with the SAM for assembling Tom40 and the receptor units of the TOM complex [9]. In contrast, the insertion of α-helical proteins depend on the action of Mim1 but the molecular details of the complex and the mechanism remain to be established [72].

The plant SAM complex is different from the one in yeast, as only Sam50 could be identified. In general, one or two Sam50 orthologues have been found in all plant species except in *G.max* (4). However, it is suggested that Sam35 and Sam37 are replaced by Metaxin [73], which can be identified in all plant species. Remarkably, we could not find a group of orthologues for Mim1 in any of the analyzed plant species (Additional files 2 and 4).

### The translocation system in the chloroplast envelope membranes

The preproteins are recognized by the TOC complex [9]. Toc64 recognizes Hsp90 loaded with preproteins [74], while Toc159 and Toc34 bind to the precursor proteins and transfer them to the pore-forming unit, Toc75 [9]. Toc64 is also part of an IMS complex with Toc12, an IMS localized Hsp70 and Tic22 [75]. However, this complex is under debate [76] and thus, the process of preprotein transfer from the outer to the inner membrane translocon complex remains largely unknown.

Logically, the analysis of the translocation system in chloroplasts is not based on a set from yeast but from *A. thaliana*. In *A. thaliana,* the Toc159 family consists of four different genes: atToc159 (Additional file 9), atToc132, atToc120 and atToc90, whereas our search for orthologues in tomato yielded five; two (Solyc01g080780 and Solyc11g043010) of which could not be assigned to a specific family member (Figure 4c and Additional file 9). The latter orthologues in tomato contains two shorter DUF3406 domains, while the other (Solyc01g080780) showed the same domain architecture as atToc159. Further, the orthologue of Toc90 (Solyc07g007650) has no “AIG1 DOMAIN”. Interestingly, Toc159 is encoded by relatively higher number of genes in *S. tuberosum* (11), *Z. mays* (7) and *P. trichocarpa* (8). Toc34 has two orthologues in Arabidopsis (atToc33 and atToc34), and two orthologues have been found in tomato (Additional file 9), both corresponding to atToc34. The other receptor unit, Toc64 has equal numbers of orthologues (3) in both *A. thaliana* and tomato. However, Toc64-I and OM64 are known not to be involved in protein translocation. In line, the C-terminal “TPR_1 DOMAIN2” does not exist in all found plant orthologues. Moreover, we observed a higher number of genes encoding Toc64 in *G. max* (7) and *P. trichocarpa* (6).

The channel forming Toc75 possess more orthologues in Arabidopsis (3) than in tomato (2). Remarkably, one of the Arabidopsis orthologue (atToc75-IV) does not have the “Surface antigen variable number repeat DOMAIN” whereas one tomato orthologue (Solyc06g076360) possess the same domain twice. Moreover, Toc12 is encoded by one gene in Arabidopsis and two in tomato, which otherwise is absent from monocots except *S. bicolor*.

In the inner membrane, Tic110, Tic62, Tic55, Tic40, Tic32, Tic21 and Tic20 constitute the TIC complex. At present, Tic110, Tic20 and Tic21 are discussed as channel forming proteins for preprotein translocation [9], while Tic62, Tic55 and Tic32 are assumed to regulate the translocon activity [77]. The energy for the translocation is fuelled by stromal chaperones Hsp70 and Hsp93 and the co-chaperone Tic40 [78]. The recently described Sec system is composed of SecY2 and SecA2 [11] and might be involved in integration or translocation of either stromal targeted or plastome encoded inner envelope proteins.

With exception of Tic32, all TIC components have the same number of orthologues in both Arabidopsis and tomato. Tic22 possess 3 and Tic20 has 5 orthologues in each of the two plants species. One of the sub-types, Tic20-I has two orthologues (AT1G04940, AT1G04945) in Arabidopsis and three in tomato (Solyc05g054720, Solyc04g076740, Solyc01g074020). However, AT1G04945 has three different splice forms (i1: 367 aa; i2: 392; i3: 644 aa) and the longest isoform (i3) extends over the neighboring gene (AT1G04940). Moreover it also contains a zinc finger-HIT domain besides the characterizing Tic20 domain whereas the tomato orthologue (Solyc01g074020) carries only the zinc finger-HIT domain and the other two orthologues have Tic20 domains. We could not detect any orthologue for Tic20-IV (AT4G03320) in tomato. The search in other plant species depicted that Tic20 is represented by higher numbers of genes in *Z. mays* and *G. max*.

Sec complexes exist in the thylakoid and inner envelope membrane, respectively. The inner envelope cpSecA2 is represented by two genes in Arabidopsis (AT1G21650 and AT1G21651), and two in tomato (Solyc11g005020 and Solyc11g005040 + Solyc11g005030; as discussed above).

### The translocation system in the thylakoid membranes

Proteins destined for thylakoid lumen are transported via cpSec and cpTat pathways [79]. Twin-arginine translocon (Tat) substrates are recognized by Hcf6 and cpTatC. After formation, this complex recruits Tha4, and the Hcf6-cpTatC-Tha4 forms the transient translocon [80]. The second path is similar to the bacterial Sec system, where cpSecA binds to the signal sequence in the stroma and guides it through the cpSecYE channel [80]. Thylakoid membrane proteins follow either spontaneous mechanism or require cpSRP for insertion. Unlike the cytoplasmic SRP complex, cpSRP has only two proteinaceous subunits - cpSRP54 and cpSRP43 [81], which post-translationally transport light harvesting proteins (LHCP) [82]. The transport complex is recognized by the thylakoid membrane receptor cpFtsY [81].

As expected, the complexes involved in import of proteins in or across the thylakoid membrane showcase a high degree of conservation. Almost all components are encoded by one orthologue in both *A. thaliana* and *S. lycopersicum*. However, several components could not be identified in certain plant species, e.g. cpSecE in tomato, *V. vinifera* and all monocots, cpSRP43 in *V. vinifera* or cpFTsY in *Z.mays*. Closer inspection of the components revealed that cpSRP43 in tomato (Solyc02g087400) carries an additional “Chromo DOMAIN” in contrast to its Arabidopsis orthologue Additional file 9).

## Conclusions

In summary, our results clearly depict a higher overlap of Arabidopsis translocation machineries with other plants than with yeast. However, all central components of the targeting and translocation machinery in the ER- and peroxisomal membranes were identified. The complexity of the translocation systems in plants largely exceeds the one in yeast. For example, we identified more than one orthologue for 62% of all the factors in *A. thaliana* and 57% of all the factors in tomato. A similar picture can be drawn for the TOC complex. On the contrary, most peroxins, TIC and thylakoid translocon components are only encoded by a single gene, which suggests that the regulation of these translocation systems is manifested by transcriptional, translational or post-translational regulation rather than by (co-)orthologue-dependent functional diversification.

For mitochondria we can conclude that the different translocation systems exist, and that the pore forming proteins are largely conserved, while many assisting proteins like receptors or assembly factor are largely diverse and could only be identified on the base of the model system *A. thaliana.* However, the outer membrane protein insertion system of mitochondria appears to be distinct between plants and yeast.

During evolution, most of the plants had undergone lineage specific duplications – both before and after splitting from each other but in most cases the complexity in the translocation machinery seems to stay stable. This high architectural conservation of protein translocation in plants can be explained by the selective pressure as consequence of the importance of the process for cellular fate and by the large number of complex components interacting with each other. The split of the evolutionary branch leading to Arabidopsis and tomato occurred after the whole genome duplication in a common ancestor approximately 140 Myr ago. Subsequently, Arabidopsis had two additional lineage specific genome duplication events, whereas tomato and potato had a single genome triplication [17]. This could be one reason for the different number of orthologues for specific factors.

Thus, we provide evidence for an overall conservation of the translocation machinery and describe the inventory in 14 different plant species, which might guide future research of plant protein import.

## Methods

### Database composition

Literature search for protein translocation machineries of different organelles was performed based on the two-model systems yeast and Arabidopsis*.* The referred literature is cited in the introduction. For all identified translocation factors in yeast and Arabidopsis the corresponding protein sequences were extracted by submitting the accession IDs to the respective databases (*S. cerevisiae*- April 2012, http://www.yeastgenome.org and *A. thaliana*- TAIR10, http://www.arabidopsis.org).

### Orthologue search

To identify orthologues of translocon components in tomato, complete set of nucleotide sequences with their respective functional annotation and the protein sequences of *S. cerevisiae* (April 2012, http://www.yeastgenome.org)*, A. thaliana* (TAIR10, http://www.arabidopsis.org) and *S. lycopersicum* (ITAG2.3, http://solgenomics.net) were downloaded. PGAP (**p**an **g**enome **a**nalysis **p**ipeline) was used to cluster protein sequences of these three species in their respective orthologous groups [83]. For this PGAP has implemented an algorithm based on the combination of InParanoid and MultiParanoid (−−method MP). The input files of PGAP had to fulfill the following criteria: (i) entries showing 3:1 relation between the coding sequence (CDS) and protein sequence lengths were considered for evaluation; (ii) same amount of CDS to protein sequences for each annotated gene; (iii) identifier for each sequence have to be unique. Terminally, by using the parameter for clustering and pangenome creation (−−cluster; --pangenome) PGAP generates the predicted orthologous groups for Arabidopsis, tomato and yeast.

In parallel, orthologue search amongst the translocon components of *S. cerevisiae* (SGD version 2011, http://yeastgenome.org) and 14 plant species was also performed using OrthoMCL [84] to span orthologous groups over more than three species in a less time consuming clustering and also compare the different predictions: (i) *A. thaliana* (TAIR10, http://www.arabidopsis.org), (ii) *B. distachyon* (bradi1.2 with GAEVAL, http://www.plantgdb.org)*,* (iii) *C. reinhardtii* (JGI v4 with GAEVAL, http://www.plantgdb.org), (iv) *G. max* (Glyma1, http://www.plantgdb.org), (v) *L. japonicus* (Lj1.0, http://www.plantgdb.org), (vi) *M. truncatula* (Mt3.5v5, http://jcvi.org), (vii) *O. sativa* (MSU Version 7.0 with GAEVAL, http://www.plantgdb.org), (viii) *P. patens* (Phypa1.6, http://phytozome.net), (ix) *P. trichocarpa* (Ptr v2.0 with GAEVAL, http://www.plantgdb.org)*,* (x) *S. lycopersicum*, (ITAG2.3, http://solgenomics.net), (xi) *S. tuberosum* (PGSC v3.4, http://solanaceae.plantbiology.msu.edu/pgsc_download.shtml), (xii) *S. bicolor* (JGI Sbi1, http://www.plantgdb.org), (xiii) *V. vinifera* (Genescope 12X, http://www.genoscope.cns.fr/spip/) and *,* (xiv) *Z. mays* (B73 RefGen v2, http://www.plantgdb.org). All genomes downloaded from PlantGDB [85] have verified annotations of genes in relation to alternative splicing and gene fusions/fissions by “gene annotation evaluation algorithm” (GAEVAL) [86]. OrthoMCL filtered away nine poor-quality sequences by our evaluation process based on the protein sequence length (<10) and percent of stop codons (marked by asterisks; >20%). The results derived from both orthologue prediction algorithms (OrthoMCL, PGAP) were used to check for consistency and automatically combined to generate the list of the translocon components in tomato. For the other plant species we had only the results of OrthoMCL.

### Factor analysis

Protein family scan from Pfam (Version 26.0) [87] was performed to predict functional domains of the protein sequences comprising the different translocons. Moreover, the order and similarity of domains of different orthologues in an individual group were analyzed automatically by using python scripts ( http://www.python.org). The name of the Pfam DOMAIN is only indicated when discussed and the description of the individual domains is deposited in the Pfam database ( http://pfam.sanger.ac.uk/).

We also downloaded next-generation sequencing (NGS) data of total RNA from *S. lycopersicum* (GEO Id: GSE33507) by using RNAseq with the 454 GS FLX Titanium and microarray data from *A. thaliana* (GEO Id: GSE5630- leaves; GSE5631- roots; GSE5632- flowers & pollen; GSE5633- shoots & stems).

For the translocation factors with at least one orthologue in both Arabidopsis and tomato, we considered seven genes up- and downstream of the translocation factor and searched for shared ‘synteny’ in that particular ‘span of genes’ by orthologue predictions. For this, we counted the number of orthologues (from both OrthoMCL and PGAP) in that span (7 upstream + 7 downstream genes) in both *Arabidopsis* and tomato. We accumulated the number of identified orthologues to gain a final syntenic score for a particular factor, which was further utilized to the claim the orthologues in Arabidopsis and tomato.

### DNA and RNA extraction for RT-PCR

Genomic DNA was isolated from young tomato leaves as previously described by Eduardo [88]. Total RNA from young leaves was isolated using the NUCLEOSPIN RNA II kit (Macherey-Nagel) following manufacturer’s instructions. First-strand cDNA was synthesized from 500 ng of RNA, using the RevertAid Reverse transcriptase (Thermo Scientific) with an oligo-dT primer following the manufacturer’s protocol. A no-RT control sample was also included, adding ddH_2_O instead of reverse transcriptase, to check for DNA contamination in cDNA samples.

PCR reaction was performed using 5 ng of cDNA, 1μM of primer, 1X *Taq* buffer, 0.2 mM dNTP mix and 0.2 μl *Taq* polymerase (60 μg/ μl) in a total volume of 20 μl. Primers used in the experiment were designed using Primer3 software ( http://primer3.sourceforge.net/) [89], whereas the forward primer (“TTTCGCTGAAGAGATGCAGA”) binds at the last exon of Solyc11g005040 and the reverse primer (“TTGCATTTTCCACCACAGAA”) binds at the first exon of Solyc11g00530 (Additional file 5).

## Competing interests

This is a manuscript of the SPOT-ITN consortium ( http://www.spot-itn.eu).

## Authors’ contributions

PP carried out the literature survey on protein translocation machineries and performed the experimental part. SS and AB performed computational analysis. SS was involved in analyzing the *in silico* results. ES conceptualized, designed and headed the project. ES, SS and PP were involved in writing the manuscript. KDS, OM and SF helped in critically analyzing the subject and helped in the final revision of the manuscript. All authors read and approved the final manuscript.

## Supplementary Material

Additional file 1: **List of references for each factor of the respective translocation machinery from the literature-based search.** The first column gives the compartment and the second the translocation factor. The following columns (3 – 6) represent the reference, where the factor was either confirmed experimentally or predicted as homologous to a translocation factor. The last column gives the information if references in the reference were used. Click here for file

Additional file 2: **The number of orthologues of translocation factors identified via Inparanoid and OrthoMCL.** The number of orthologues of translocation factors identified via InParanoid and OrthoMCL. The first column represents the compartment and second- the translocation factor. The following columns (3 – 17) represent the number of detected orthologues in the different species: *S. cerevisiae*, *A. thaliana*, *S. lycopersicum*, *S. tuberosum*, *G. max*, *B. distachyon*, *L. japonicus*, *M. truncatula*, *O. sativa*, *P. patens*, *C. reinhardtii, P. trichocarpa*, *S. bicolor*, *V. vinifera* and *Z. mays*. In column 3–5 (*S. cerevisiae*, *A. thaliana* and *S. lycopersicum*), the first number is the ‘number of orthologues detected by OrthoMCL’ and the second number is the ‘number of orthologues detected via Inparanoid’. Click here for file

Additional file 3: **The orthologues of translocation factors identified via Inparanoid.** The identifiers of the orthologues of translocation factors identified via InParanoid. The first column gives the compartment and the second the translocation factor. The following columns represent the identifier of the orthologues in *S. cerevisiae*, *A. thaliana* and *S. lycopersicum*. Click here for file

Additional file 4: **The orthologues of translocation factors identified via OrthoMCL.** The identifier of orthologues of translocation factors identified via OrthoMCL. The first column gives the compartment and the second the translocation factor. The following columns (3 – 17) give the identifier of detected orthologues in the different species: *S. cerevisiae*, *A. thaliana*, *S. lycopersicum*, *S. tuberosum*, *G. max*, *B. distachyon*, *L. japonicus*, *M. truncatula*, *O. sativa*, *P. patens*, *C. reinhardtii, P. trichocarpa*, *S. bicolor*, *V. vinifera* and *Z. mays*. Click here for file

Additional file 5: **Table of components of the translocon at the ER surface.** Given is the general path (column 1), the central complex name (column 2), the standard name of the component (column 3), the accession number for the yeast (column 4), A. thaliana (column 5) and tomato (column 6) gene coding for the component and the amino acid length of the yeast (column 7), A. thaliana (column 8) and tomato protein (column 9). NF no factor detected. *Same orthology group as Sbh1p, + depicts the correlation via syntenic analysis. # signifies correlation on the basis of expression pattern. Click here for file

Additional file 6 **Table of components of the ERAD system.** Given is the central complex name (column 1), the name of the component (column 2), the accession number for the yeast (column 3), A. thaliana (column 4) and tomato (column 5) gene coding for the component and the amino acid length of the yeast (column 6), A. thaliana (column 7) and tomato protein (column 8). NF no factor detected with the settings described in materials and methods; * [36], + depicts the correlation via syntenic analysis. # signifies correlation on the basis of expression pattern. Click here for file

Additional file 7: **Table of peroxisomal components involved in protein translocation.** Given is the central complex name (column 1), the name of the component (column 2), the accession number for the yeast (column 3), A. thaliana (column 4) and tomato (column 5) gene coding for the component and the amino acid length of the yeast (column 6), A. thaliana (column 7) and tomato protein (column 8). NF no factor detected with the settings described in materials and methods. * [51]; ** [54]; ***same orthology group as Ubc4, + depicts the correlation via syntenic analysis, # signifies correlation on the basis of expression pattern. Click here for file

Additional file 9: **Table of components involved in protein translocation in chloropalsts.** Given is the pathway and the name of the protein family (column 1), the name of the component (column 2), the accession number for the A. thaliana (column 3) and tomato (column 4) gene coding for the component and the amino acid length of the A. thaliana (column 5) and tomato protein (column 6). NF no factor detected with the settings described in materials and methods *Please note, that all orthologues for the entire protein family are listed and not in the direct relation to each other. Click here for file

Additional file 10: **Schematic representation of RT-PCR experiment for cpSecA2 in tomato.** We designed a forward primer (Fp) from the exonic region (3^′^ end) of Solyc11g005040 and a reverse primer (Rp) from the exonic region of Solyc11g005030. As a result, we got an amplification of ~400bp in both genomic DNA and cDNA, which is the distance between both primers including the 140 bp between the two annotated genes (Solyc11g005040 and Solyc11g005030). Click here for file

Additional file 11: **Expression levels of genes coding for ER and ERAD translocon components in tomato and Arabidopsis.** Expression level of genes coding for ER **(a)** and ERAD **(b)** translocon components in tomato and Arabidopsis. The normalized microarray data from *A. thaliana (*left, multiplication factor 100) and the NGS data from *S. lycopersicum* (right, multiplication factor 1000) are shown for the tissues: leaves (LE), flower and pollen (F & P), shoots and stems (S & S) and roots (RO). The arrangement of the expression patterns correlates with the orthologues found for the different factors of the ER translocation machinery. In general, we made a few interesting observations: (i) RNAseq data for LE and F & P in tomato are extremely low for most of the translocation machineries in the ER and ERAD; (ii) components possessing >1 orthologues in both plant species have in general one of their orthologues with higher expression than the others in the respective plant (e.g. ER: Srp54, Srp72, Sec61; ERAD: Ubc6), (iii) Only for a few components possessing >1 orthologue in tomato and Arabidopsis all orthologues show either high expression (Srp102, Ubc7) or low expression (Hrd1). Click here for file

Additional file 12 **Expression levels of genes coding for peroxisomal translocation components in tomato and Arabidopsis.** Expression level of genes coding for peroxisomal translocation components in tomato and Arabidopsis. The peroxisomal translocation factors and their orthologues in tomato (right) and Arabidopsis (left) are assigned according to their normalized microarray data (*A. thaliana,* multiplication factor 100) and NGS data (*S. lycopersicum*, multiplication factor 1000) in different tissues: leaves (LE), flower and pollen (F & P), shoots and stems (S & S) and roots (RO). Similar to the expression of the ER translocation machinery (Additional file 11), we notified low expression in leaves and flower & pollen (LE and F & P) for the peroxisomal translocation machinery in tomato. On the contrary, there is a high expression for Ubc4, Ubc5 and Pex4 for all tissues in tomato, while Pex5, Pex12, Pex10, Pex2 and Pex6 are expressed at low levels in tomato in all tissues examined. Remarkably, the orthologue from Pex15 and Pex19 in tomato correlates more to one of their orthologues in Arabidopsis (Pex19p: AT3G03490/ Solyc06g060720; Pex15p: AT3G10572/ Solyc12g017470). Click here for file

Additional file 13: **Expression levels of genes coding for mitochondrial translocation components in tomato and Arabidopsis.** Expression level of genes coding for mitochondrial translocation components in tomato and Arabidopsis. For leaves (LE), flower and pollen (F & P), shoots and stems (S & S) and roots (RO) the normalized microarray data from *A. thaliana* (left, multiplication factor 100) and NGS data from *S. lycopersicum* (right, multiplication factor 1000) are shown. The expression profiles of the mitochondria translocation machinery are arranged according to their orthologues. We observed low expression in leaves and flower & pollen (LE and F & P) of components in tomato, which is also seen for in the ER and peroxisome compartments (Additional files 11, 12). For tomato, we observed lower expression for orthologs to Tom70, OM64, Tim50, Oxa1, Pam18 and Metaxin under all conditions. Only for Mia40, Tim21 and ERV1 there is no orthologue with high expression in Arabidopsis or tomato. Remarkably, Tom6 and Tim13 are the unique factors in tomato, which had a high expression in leaves. Click here for file

Additional file 14: **Expression levels of genes coding for plastidic translocation components in tomato and Arabidopsis.** Expression level of genes coding for plastidic translocation components in tomato and Arabidopsis. For leaves (LE), flower and pollen (F & P), shoots and stems (S & S) and roots (RO) the normalized microarray data from *A. thaliana* (left, multiplication factor 100) and NGS data from *S. lycopersicum* (right, multiplication factor 1000) are shown. The expression profiles of the chloroplast translocation factors are arranged according to their orthologues. From the expression pattern of translocation components of both mitochondria and chloroplast, we observed lower expression in leaves and flower & pollen (LE and F & P) of components in tomato, which is same in the ER and peroxisome compartments. We observed extremely low expression for cpSecA2, Toc75-IV, Tic32-IVa (AT4G23430) and Tic20-I in Arabidopsis compared to other translocon components. In tomato we notified high expression for Toc12, Tic20-V, Tic21 and Tha4 (RO and S & S). Remarkably, Tic32-IVa and Toc64-I are highly expressed in tomato leaves, whereas Toc12 (Solyc06g068500) is the only gene of putative chloroplast translocon components expressed high in flower and pollen (F & P). Click here for file

Additional file 15: **The shared synteny analysis of Arabidopsis and tomato protein translocation factors.** Shared synteny between translocation factors of tomato and Arabidopsis. The first column gives the compartment and the second the translocation factor. The third column consist the AGI of Arabidopsis and the fourth column the identifier of the SGN for tomato. The last three columns (5– 7) give the syntenic score for OrthoMCL (column 5); InParanoid (column 6) and both methods (column 7). The syntenic score is calculated for each pair of orthologues in Arabidopsis and tomato for the factor of interest. Number of orthologues for seven genes upstream and seven genes downstream of the genes of interest in Arabidopsis and tomato are listed as syntenic score. Click here for file

Additional file 8: **Table of components involved in protein translocation.** Given is the central complex name (column 1), the name of the component (column 2), the accession number for the yeast (column 3), A. thaliana (column 4) and tomato (column 5) gene coding for the component and the amino acid length of the yeast (column 6) A. thaliana (column 7) and tomato protein (column 8). NF no factor detected with the settings described in materials and methods. * [63]; ** same orthology group as Pam18, + depicts the correlation via syntenic analysis, # signifies correlation on the basis of expression pattern. Click here for file
