# Supplementary material for: The protein translocation systems in plants – composition and variability on the example of Solanum lycopersicum
Source: BMC Genomics. 2013 Mar 18;14:189. doi: 10.1186/1471-2164-14-189 (PMC3610429; doi:10.1186/1471-2164-14-189)
Supplement: Additional file 5: — Table of components of the translocon at the ER surface. Given is the general path (column 1), the central complex name (column 2), the standard name of the component (column 3), the accession number for the yeast (column 4), A. thaliana (column 5) and tomato (column 6) gene coding for the component and the amino acid length of the yeast (column 7), A. thaliana (column 8) and tomato protein (column 9). NF no factor detected. *Same orthology group as Sbh1p, + depicts the correlation via syntenic analysis. # signifies correlation on the basis of expression pattern. [file 1471-2164-14-189-S5.docx]

**Additional file 5 Table of components of the translocon at the ER surface.**

| **PATH** | | **FACTOR** | **YEAST** | ***A. thaliana*** | **TOMATO** | **Y-AA** | **A-AA** | **T-AA** |
| --- | --- | --- | --- | --- | --- | --- | --- | --- |
| Co-translational targeting | SRPs | Srp54p | YPR088C | AT1G48900^#^ | Solyc12g042740 | 541 | 495 | 500 |
|  |  |  |  | AT5G49500 | Solyc03g116810^#^ |  | 497 | 590 |
|  |  |  |  | AT1G15310 |  |  | 479 |  |
|  |  | Srp72p | YPL210C | AT1G67650 | Solyc11g062270^#^ | 640 | 651 | 664 |
|  |  |  |  | AT1G67680^#^ | Solyc01g047590 |  | 664 | 404 |
|  |  |  |  |  | Solyc10g033560 |  |  | 256 |
|  |  |  |  |  | Solyc00g287330 |  |  | 182 |
|  |  | Sec65p | YML105C | AT1G48160 | Solyc08g082740 | 273 | 145 | 213 |
|  |  | Srp14p | YDL092W | NF | NF | 146 |  |  |
|  |  | Srp68p | YPL243W | NF | NF | 599 |  |  |
|  |  | Srp21p | YKL122C | NF | NF | 167 |  |  |
|  |  | Srp7p | NF | NF | NF |  |  |  |
|  | SR | SRα/ SRP101 | YDR292C | AT4G30600 | Solyc12g009990 | 621 | 634 | 619 |
|  |  | SRβ/ SRP102 | YKL154W | AT5G05670 | Solyc03g112450 | 244 | 260 | 261 |
|  |  |  |  | AT2G18770 | Solyc11g043150 |  | 260 | 261 |
| Post-transl. trans. | Sec62/63 complex | Sec62p | YPL094C | AT3G20920 | Solyc11g065690 | 274 | 365 | 342 |
|  |  |  |  |  | Solyc01g008470 |  |  | 359 |
|  |  | Sec63p | YOR254C | AT4G21180 | Solyc09g009180 | 663 | 661 | 686 |
|  |  |  |  | AT1G79940 | Solyc03g007610 |  | 687 | 715 |
|  |  | Sec71p/ Sec66p | YBR171W | NF | NF | 206 |  |  |
|  |  | Sec72p/ Sec67p | YLR292C | NF | NF | 193 |  |  |
|  |  | atTPR7 | NF | AT5G21990 | Solyc06g073840 |  | 554 | 589 |
| Translocation | Sec61 complex | Sec61p | YLR378C | AT1G78720^+#^ | Solyc10g007390^+#^ | 480 | 475 | 475 |
|  |  |  |  | AT2G34250 |  |  | 475 |  |
|  |  |  |  | AT1G29310^+^ | Solyc02g072130^+^ |  | 475 | 475 |
|  |  | Sbh1p | YER087C-B | AT3G60540 | Solyc03g116990 | 81 | 81 | 109 |
|  |  |  |  | AT2G45070 | Solyc06g010060 |  | 82 | 112 |
|  |  |  |  | AT5G60460 |  |  | 109 |  |
|  |  | Sbh2p* | YER019C-A |  |  | 88 |  |  |
|  |  | Sss1p | YDR086C | AT5G50460 | NF | 80 | 69 |  |
|  |  |  |  | AT4G24920 |  |  | 69 |  |
|  |  |  |  | AT3G48570 |  |  | 69 |  |
|  |  | Ssh1p | YBR283C | NF | NF | 490 |  |  |
| Tail-anchored proteins | Get complex | Sgt2 | YOR007C | AT4G08320 | Solyc11g012900 | 346 | 427 | 423 |
|  |  | Get4 | YOR164C | AT5G63220 | Solyc08g015990 | 312 | 324 | 328 |
|  |  |  |  |  | Solyc12g017820 |  |  | 325 |
|  |  | Get3 | YDL100C | AT1G01910^+^ | Solyc01g091880^+^ | 354 | 353 | 360 |
|  |  |  |  |  | Solyc10g017810 |  |  | 363 |
|  |  |  |  |  | Solyc05g050480 |  |  | 405 |
|  |  |  |  |  | Solyc05g050490 |  |  | 762 |
|  |  | Get5 | YOL111C | NF | NF | 212 |  |  |
|  |  | Get1 | YGL020C | NF | NF | 235 |  |  |
|  |  | Get2 | YER083C | NF | NF | 285 |  |  |
| Given is the general path (column 1), the central complex name (column 2), the standard name of the component (column 3), the accession number for the yeast (column 4), *A. thaliana* (column 5) and tomato (column 6) gene coding for the component and the amino acid length of the yeast (column 7), *A. thaliana* (column 8) and tomato protein (column 9). NF … no factor detected. *Same orthology group as Sbh1p, ^+^ depicts the correlation via syntenic analysis. ^#^ signifies correlation on the basis of expression pattern. | | | | | | | | |
