# Supplementary material for: The protein translocation systems in plants – composition and variability on the example of Solanum lycopersicum
Source: BMC Genomics. 2013 Mar 18;14:189. doi: 10.1186/1471-2164-14-189 (PMC3610429; doi:10.1186/1471-2164-14-189)
Supplement: Additional file 9: — Table of components involved in protein translocation in chloropalsts. Given is the pathway and the name of the protein family (column 1), the name of the component (column 2), the accession number for the A. thaliana (column 3) and tomato (column 4) gene coding for the component and the amino acid length of the A. thaliana (column 5) and tomato protein (column 6). NF no factor detected with the settings described in materials and methods *Please note, that all orthologues for the entire protein family are listed and not in the direct relation to each other. [file 1471-2164-14-189-S9.docx]

**Additional file 9 Table of components involved in protein translocation in chloroplasts.**

| **PATH** | | **FACTOR** | ***A. thaliana**** | **TOMATO** | **A-AA** | **T-AA** |
| --- | --- | --- | --- | --- | --- | --- |
| TOC complex in OE | Toc159 | atToc159 | AT4G02510 | Solyc01g111240 | 1503 | 1341 |
|  |  | atToc132 | AT2G16640 | Solyc09g074940 | 1206 | 1409 |
|  |  | atToc120 | AT3G16620 |  | 1089 |  |
|  |  |  |  | Solyc01g080780 |  | 1103 |
|  |  |  |  | Solyc11g043010 |  | 728 |
|  |  | atToc90 | AT5G20300 | Solyc07g007650 | 793 | 543 |
|  | Toc34 | atToc33 | AT1G02280 | NF | 297 |  |
|  |  | atToc34 | AT5G05000 | Solyc03g095220 | 313 | 259 |
|  |  |  |  | Solyc05g052160 |  | 301 |
|  | Toc75 | atToc75-III | AT3G46740 | Solyc06g076360 | 818 | 812 |
|  |  | atToc75-IV | AT4G09080 | NF | 396 |  |
|  |  | atToc75-V/AtOEP80 | AT5G19620 | Solyc11g062070 | 732 | 698 |
|  |  |  |  | Solyc10g079290 |  | 703 |
|  | Toc64 | atToc64-I | AT1G08980 | Solyc10g086170 | 425 | 426 |
|  |  | atToc64-III | AT3G17970 | Solyc03g118690 | 589 | 590 |
|  |  | atToc64-V | AT5G09420 | Solyc10g047300 | 603 | 598 |
|  | Toc12 | atToc12 | AT1G80920 | Solyc06g068500 | 163 | 162 |
|  |  |  |  | Solyc03g117590 |  | 161 |
| TIC complex in IE | Tic22 | atTic22-IV | AT4G33350 | Solyc04g079000 | 268 | 293 |
|  |  | atTic22-III | AT3G23710 | Solyc09g092530 | 313 | 263 |
|  |  | Tic22-like | AT5G62650 | Solyc03g007620 | 529 | 462 |
|  | Tic20 | atTic20-I | AT1G04940 | Solyc04g076740 | 274 | 271 |
|  |  |  | AT1G04945 | Solyc05g054720 | 367 | 271 |
|  |  |  |  | Solyc01g074020 |  | 373 |
|  |  | atTic20-IV | AT4G03320 | NF | 284 |  |
|  |  | atTic20-II | AT2G47840 | Solyc11g008980 | 208 | 204 |
|  |  | atTic20-V | AT5G55710 | Solyc12g015680 | 209 | 197 |
|  | Tic21 | atTic21 | AT2G15290 | Solyc01g107640 | 296 | 283 |
|  | Tic110 | atTic110 | AT1G06950 | Solyc09g031780 | 1016 | 1005 |
|  | Tic40 | atTic40 | AT5G16620 | Solyc11g020300 | 447 | 443 |
|  | Tic62 | atTic62 | AT3G18890 | Solyc10g051110 | 641 | 734 |
|  | Tic55 | atTic55 | AT2G24820 | Solyc12g096550 | 539 | 546 |
|  | Tic32 | atTic32-IVa | AT4G23430 | Solyc03g025410 | 322 | 314 |
|  |  |  | AT4G23420 | Solyc03g025400 | 333 | 317 |
|  |  |  |  | Solyc03g025390 |  | 314 |
|  |  | atTic32-IVb | AT4G11410 | Solyc08g081200 | 317 | 315 |
| IM Sec Pathway | | cpSecA2 | AT1G21650 | Solyc11g005020 | 1051 | 1288 |
|  |  |  | AT1G21651 | Solyc11g005030 | 811 | 364 |
|  |  |  |  | Solyc11g005040 |  | 383 |
|  |  | cpSecY2 | AT2G31530 | Solyc09g065120 | 575 | 576 |
| Thylakoid Sec Pathway | | cpSecA | AT4G01800 | Solyc01g080840 | 1042 | 1018 |
|  |  | cpSecY | AT2G18710 | Solyc07g006520 | 551 | 536 |
|  |  | cpSecE | AT4G14870 | NF | 177 |  |
| TAT path-way | | Tha4 | AT5G28750 | Solyc01g097310 | 147 | 160 |
|  |  | Hcf106 | AT5G52440 | Solyc03g025310 | 260 | 375 |
|  |  | cpTatC | AT2G01110 | Solyc05g008530 | 340 | 351 |
| SRP path-way | | cpSRP43 | AT2G47450 | Solyc02g087400 | 373 | 368 |
|  |  | cpSRP54 | AT5G03940 | Solyc09g009940 | 564 | 567 |
|  |  | cpFtsY | AT2G45770 | Solyc01g091580 | 373 | 368 |
|  |  | ALB3 | AT2G28800 | Solyc11g066110 | 462 | 447 |
| Given is the pathway and the name of the protein family (column 1), the name of the component (column 2), the accession number for the *A. thaliana* (column 3) and tomato (column 4) gene coding for the component and the amino acid length of the *A. thaliana* (column 5) and tomato protein (column 6). NF … no factor detected with the settings described in materials and methods. *Please note, that all orthologues for the entire protein family are listed and not in the direct relation to each other. | | | | | | |
