# Supplementary figures and images for: The protein translocation systems in plants – composition and variability on the example of Solanum lycopersicum
Source: BMC Genomics. 2013 Mar 18;14:189. doi: 10.1186/1471-2164-14-189 (PMC3610429; doi:10.1186/1471-2164-14-189)

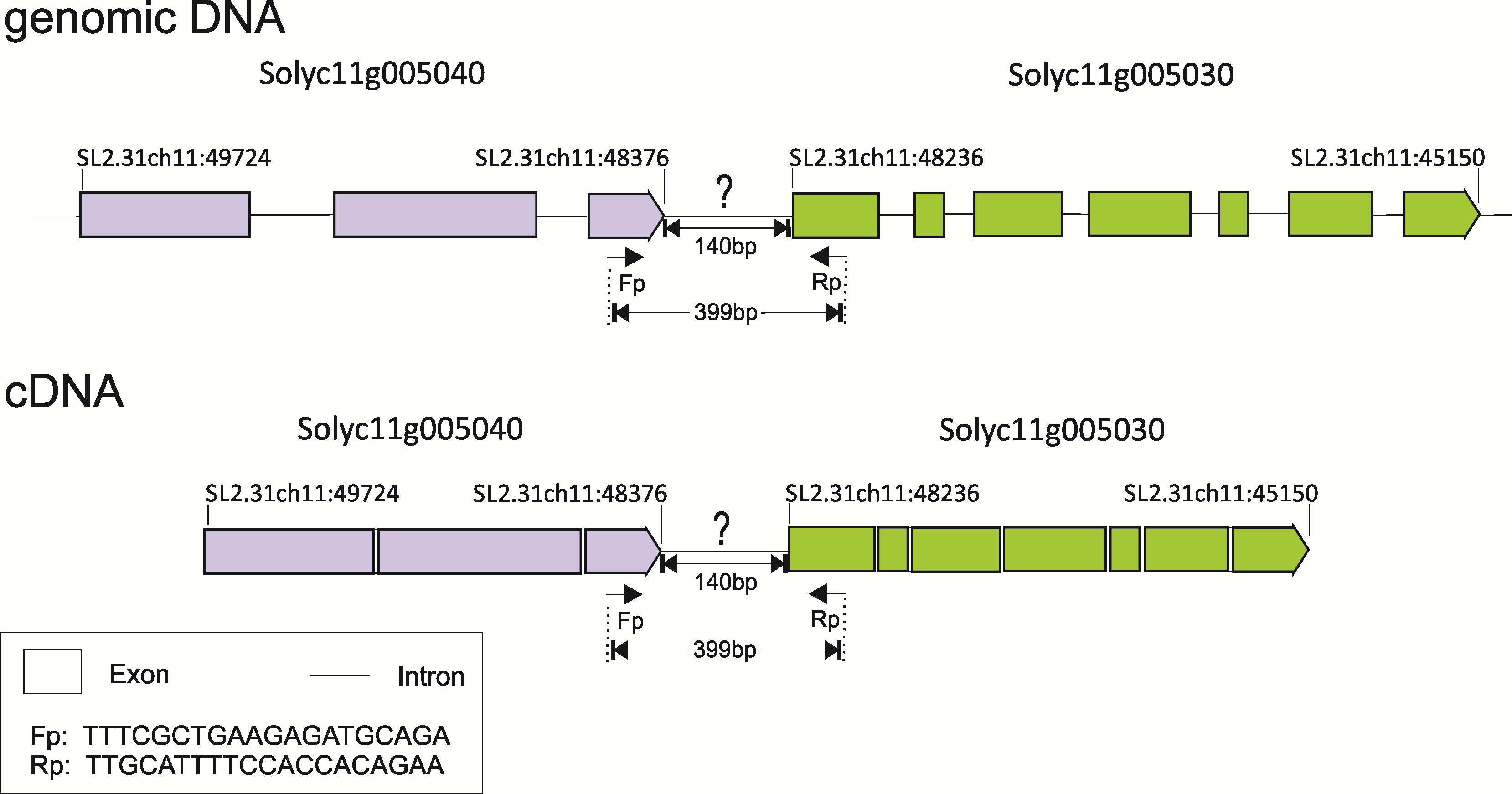


**Additional file 5**

Supplement: Additional file 10: — Schematic representation of RT-PCR experiment for cpSecA2 in tomato. We designed a forward primer (Fp) from the exonic region (3′ end) of Solyc11g005040 and a reverse primer (Rp) from the exonic region of Solyc11g005030. As a result, we got an amplification of ~400bp in both genomic DNA and cDNA, which is the distance between both primers including the 140 bp between the two annotated genes (Solyc11g005040 and Solyc11g005030). [file 1471-2164-14-189-S10.doc]

**(a)**
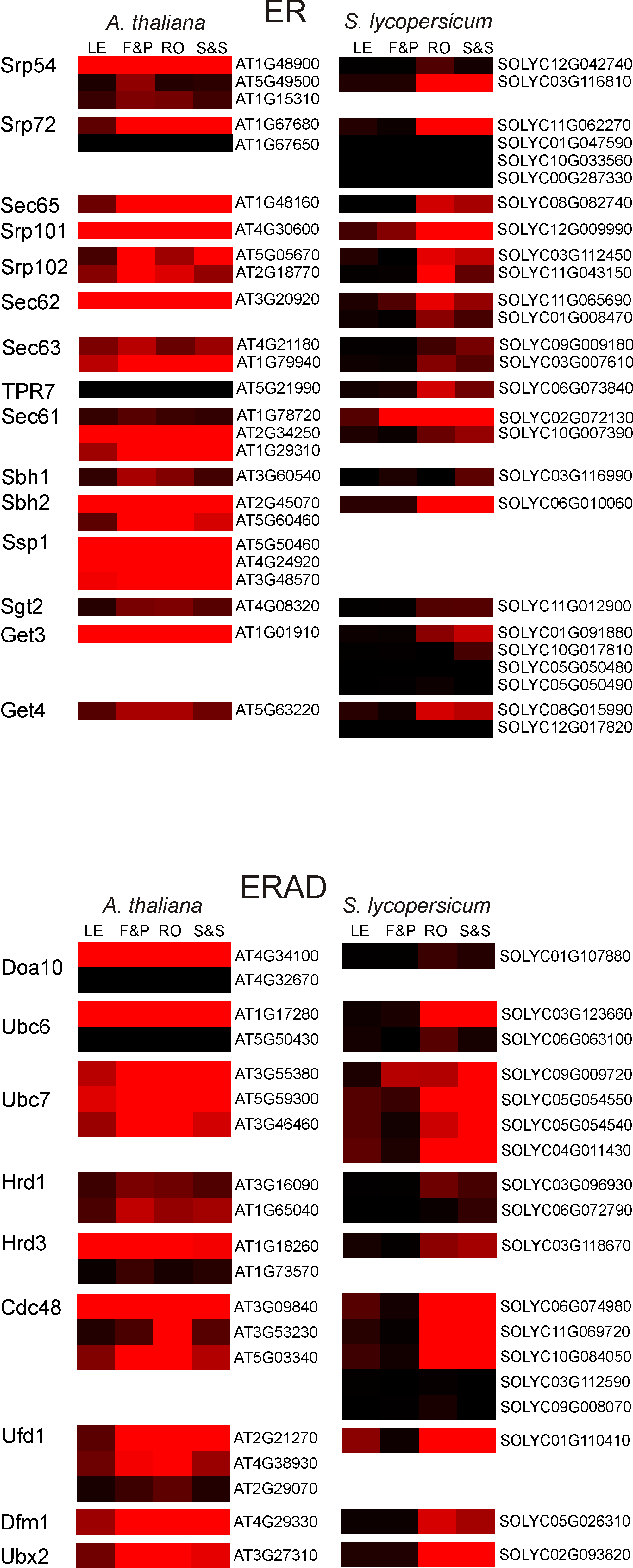

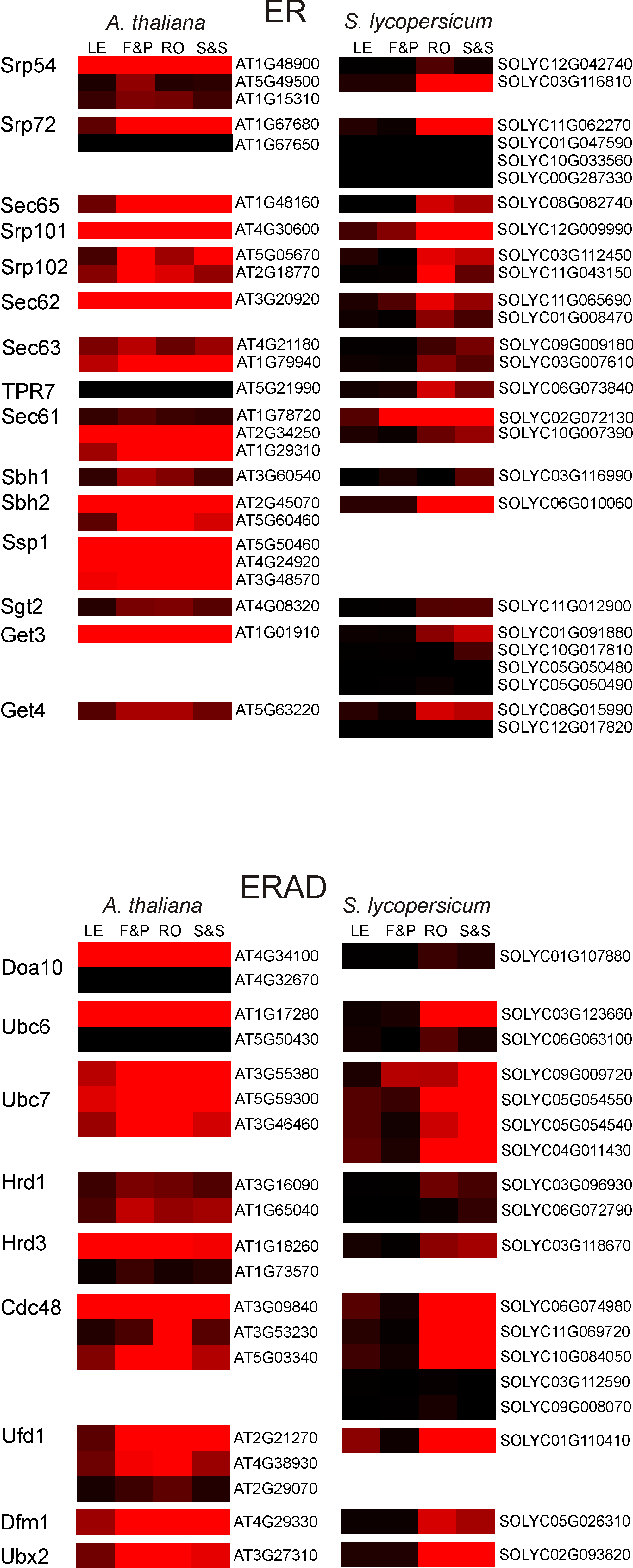


**(b)**

**Additional file 6**

Supplement: Additional file 11: — Expression levels of genes coding for ER and ERAD translocon components in tomato and Arabidopsis. Expression level of genes coding for ER (a) and ERAD (b) translocon components in tomato and Arabidopsis. The normalized microarray data from A. thaliana (left, multiplication factor 100) and the NGS data from S. lycopersicum (right, multiplication factor 1000) are shown for the tissues: leaves (LE), flower and pollen (F & P), shoots and stems (S & S) and roots (RO). The arrangement of the expression patterns correlates with the orthologues found for the different factors of the ER translocation machinery. In general, we made a few interesting observations: (i) RNAseq data for LE and F & P in tomato are extremely low for most of the translocation machineries in the ER and ERAD; (ii) components possessing >1 orthologues in both plant species have in general one of their orthologues with higher expression than the others in the respective plant (e.g. ER: Srp54, Srp72, Sec61; ERAD: Ubc6), (iii) Only for a few components possessing >1 orthologue in tomato and Arabidopsis all orthologues show either high expression (Srp102, Ubc7) or low expression (Hrd1). [file 1471-2164-14-189-S11.doc]

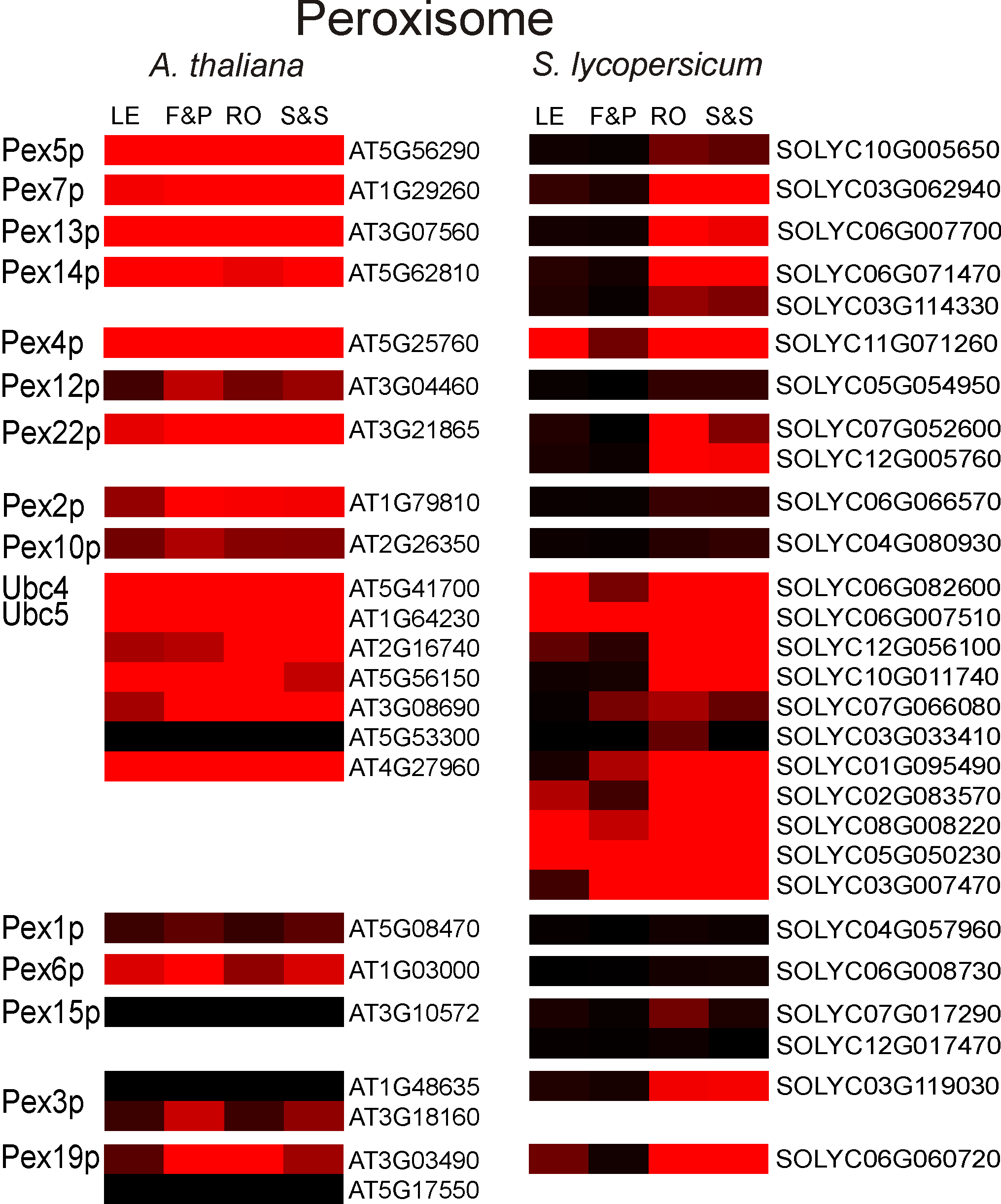


**Additional file 7**

Supplement: Additional file 12 — Expression levels of genes coding for peroxisomal translocation components in tomato and Arabidopsis. Expression level of genes coding for peroxisomal translocation components in tomato and Arabidopsis. The peroxisomal translocation factors and their orthologues in tomato (right) and Arabidopsis (left) are assigned according to their normalized microarray data (A. thaliana, multiplication factor 100) and NGS data (S. lycopersicum, multiplication factor 1000) in different tissues: leaves (LE), flower and pollen (F & P), shoots and stems (S & S) and roots (RO). Similar to the expression of the ER translocation machinery (Additional file 11), we notified low expression in leaves and flower & pollen (LE and F & P) for the peroxisomal translocation machinery in tomato. On the contrary, there is a high expression for Ubc4, Ubc5 and Pex4 for all tissues in tomato, while Pex5, Pex12, Pex10, Pex2 and Pex6 are expressed at low levels in tomato in all tissues examined. Remarkably, the orthologue from Pex15 and Pex19 in tomato correlates more to one of their orthologues in Arabidopsis (Pex19p: AT3G03490/ Solyc06g060720; Pex15p: AT3G10572/ Solyc12g017470). [file 1471-2164-14-189-S12.doc]

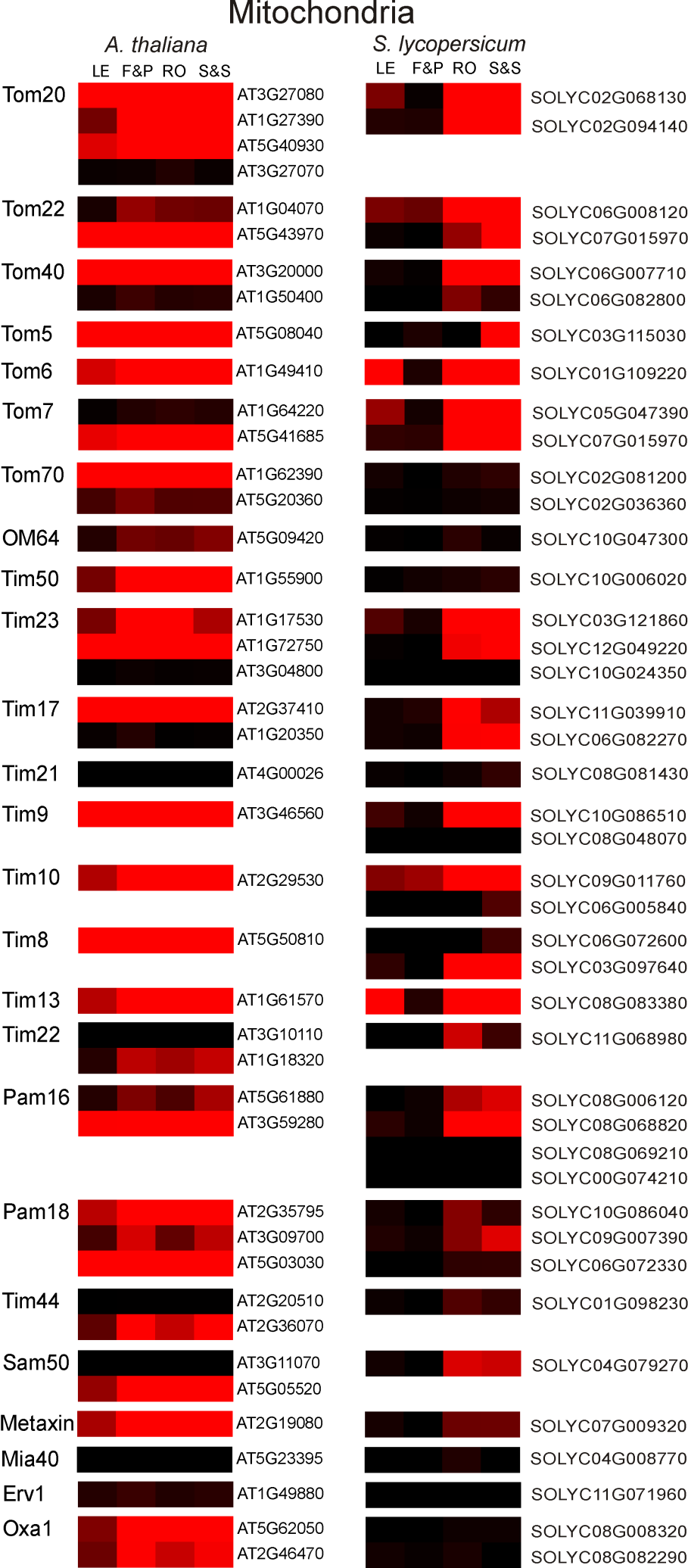


**Additional file 8**

Supplement: Additional file 13: — Expression levels of genes coding for mitochondrial translocation components in tomato and Arabidopsis. Expression level of genes coding for mitochondrial translocation components in tomato and Arabidopsis. For leaves (LE), flower and pollen (F & P), shoots and stems (S & S) and roots (RO) the normalized microarray data from A. thaliana (left, multiplication factor 100) and NGS data from S. lycopersicum (right, multiplication factor 1000) are shown. The expression profiles of the mitochondria translocation machinery are arranged according to their orthologues. We observed low expression in leaves and flower & pollen (LE and F & P) of components in tomato, which is also seen for in the ER and peroxisome compartments (Additional files 11, 12). For tomato, we observed lower expression for orthologs to Tom70, OM64, Tim50, Oxa1, Pam18 and Metaxin under all conditions. Only for Mia40, Tim21 and ERV1 there is no orthologue with high expression in Arabidopsis or tomato. Remarkably, Tom6 and Tim13 are the unique factors in tomato, which had a high expression in leaves. [file 1471-2164-14-189-S13.doc]

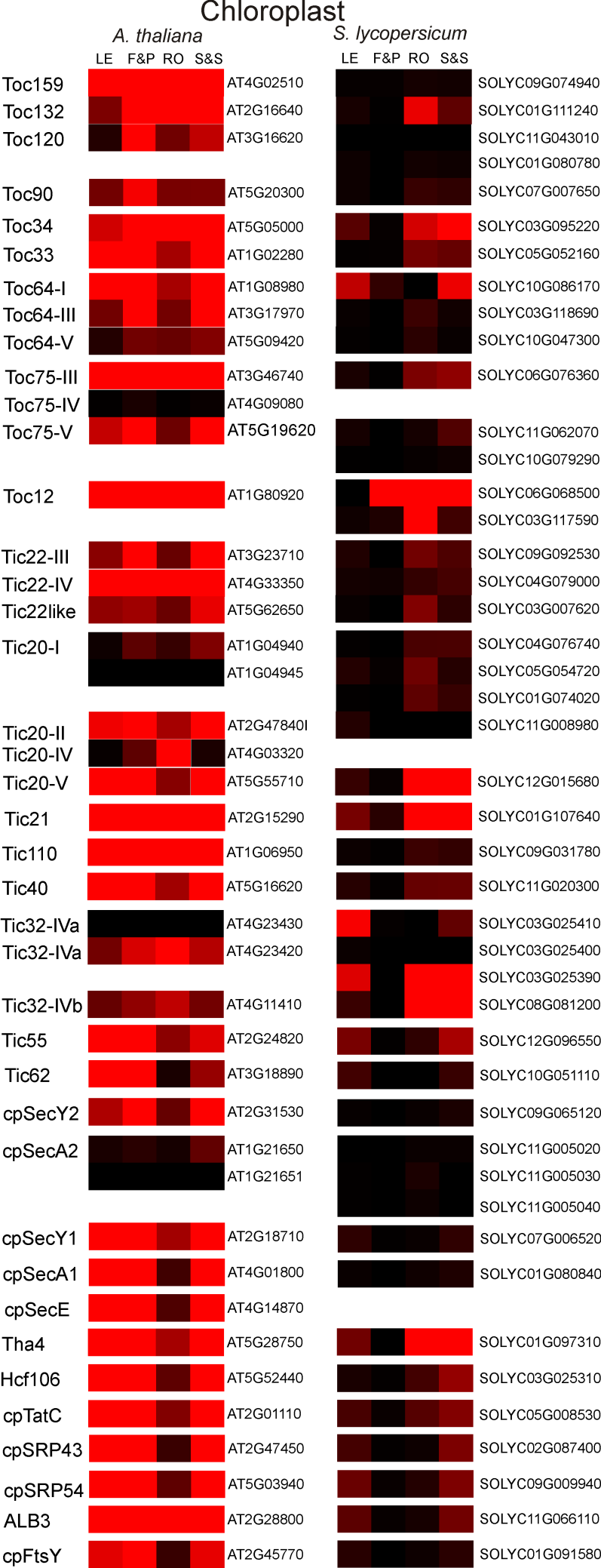


**Additional file 9**

Supplement: Additional file 14: — Expression levels of genes coding for plastidic translocation components in tomato and Arabidopsis. Expression level of genes coding for plastidic translocation components in tomato and Arabidopsis. For leaves (LE), flower and pollen (F & P), shoots and stems (S & S) and roots (RO) the normalized microarray data from A. thaliana (left, multiplication factor 100) and NGS data from S. lycopersicum (right, multiplication factor 1000) are shown. The expression profiles of the chloroplast translocation factors are arranged according to their orthologues. From the expression pattern of translocation components of both mitochondria and chloroplast, we observed lower expression in leaves and flower & pollen (LE and F & P) of components in tomato, which is same in the ER and peroxisome compartments. We observed extremely low expression for cpSecA2, Toc75-IV, Tic32-IVa (AT4G23430) and Tic20-I in Arabidopsis compared to other translocon components. In tomato we notified high expression for Toc12, Tic20-V, Tic21 and Tha4 (RO and S & S). Remarkably, Tic32-IVa and Toc64-I are highly expressed in tomato leaves, whereas Toc12 (Solyc06g068500) is the only gene of putative chloroplast translocon components expressed high in flower and pollen (F & P). [file 1471-2164-14-189-S14.doc]
