# Supplementary material for: The protein translocation systems in plants – composition and variability on the example of Solanum lycopersicum
Source: BMC Genomics. 2013 Mar 18;14:189. doi: 10.1186/1471-2164-14-189 (PMC3610429; doi:10.1186/1471-2164-14-189)
Supplement: Additional file 8: — Table of components involved in protein translocation. Given is the central complex name (column 1), the name of the component (column 2), the accession number for the yeast (column 3), A. thaliana (column 4) and tomato (column 5) gene coding for the component and the amino acid length of the yeast (column 6) A. thaliana (column 7) and tomato protein (column 8). NF no factor detected with the settings described in materials and methods. * [63]; ** same orthology group as Pam18, + depicts the correlation via syntenic analysis, # signifies correlation on the basis of expression pattern. [file 1471-2164-14-189-S8.docx]

**Additional file 8 Table of components involved in protein translocation in mitochondria.**

| **PATH** | **FACTOR** | **YEAST** | ***A. thaliana*** | **TOMATO** | **Y-AA** | **A-AA** | **T-AA** |
| --- | --- | --- | --- | --- | --- | --- | --- |
| TOM complex | Tom20 | YGR082W | NF | NF | 183 |  |  |
|  |  |  | AT1G27390* | Solyc02g068130 |  | 188 | 205 |
|  |  |  | AT3G27080* | Solyc02g094140 |  | 202 | 204 |
|  |  |  | AT3G27070* |  |  | 248 |  |
|  |  |  | AT5G40930* |  |  | 187 |  |
|  | Tom22 | YNL131W | NF | NF | 152 |  |  |
|  |  |  | AT1G04070* | Solyc06g008120 |  | 94 | 107 |
|  |  |  | AT5G43970* | Solyc03g005700 |  | 99 | 100 |
|  | Tom40 | YMR203W | AT3G20000^#^ | Solyc06g007710^#^ | 387 | 309 | 314 |
|  |  |  | AT1G50400 | Solyc06g082800 |  | 310 | 312 |
|  | Tom5 | YPR133W-A | NF | NF | 50 |  |  |
|  |  |  | AT5G08040* | Solyc03g115030 |  | 54 | 54 |
|  | Tom6 | YOR045W | AT1G49410 | Solyc01g109220 |  | 54 | 54 |
|  | Tom7 | YNL070W | NF | NF | 60 |  |  |
|  |  |  | AT1G64220 | Solyc05g047390 |  | 77 | 78 |
|  |  |  | AT5G41685 | Solyc07g015970 |  | 75 | 73 |
|  | Tom70 | YNL121C | AT1G62390 | Solyc02g081200 | 617 | 751 | 779 |
|  |  | YHR117W | AT5G20360 | Solyc02g036360 |  | 809 | 762 |
|  | OM64 | NF | AT5G09420 | Solyc10g047300 |  | 603 | 599 |
| TIM23 complex | Tim50 | YPL063W | AT1G55900 | Solyc10g006020 | 476 | 376 | 359 |
|  | Tim23 | YNR017W | AT1G17530 | Solyc03g121860 | 222 | 187 | 191 |
|  |  |  | AT1G72750 | Solyc12g049220 |  | 188 | 185 |
|  |  |  | AT3G04800 | Solyc10g024350 |  | 188 | 151 |
|  | Tim17 | YJL143W | AT2G37410 | Solyc11g039910 | 158 | 243 | 233 |
|  |  |  | AT1G20350 | Solyc06g082270 |  | 218 | 236 |
|  | Tim21 | YGR033C | NF | NF | 239 |  |  |
|  |  |  | AT4G00026* | Solyc08g081430 |  | 269 | 311 |
| Small IMS chaperones | Tim9 | YEL020W-A | AT3G46560^+#^ | Solyc10g086510^+#^ | 87 | 93 | 94 |
|  |  |  |  | Solyc08g048070 |  |  | 68 |
|  | Tim10 | YHR005C-A | AT2G29530^#^ | Solyc09g011760^#^ | 93 | 107 | 82 |
|  |  |  |  | Solyc06g005840 |  |  | 63 |
|  | Tim8 | YJR135W-A | AT5G50810^#^ | Solyc06g072600 | 87 | 77 | 79 |
|  |  |  |  | Solyc03g097640^#^ |  |  | 77 |
|  | Tim13 | YGR181W | AT1G61570 | Solyc08g083380 | 105 | 87 | 89 |
| TIM22 complex | Tim22 | YDL217C | AT3G10110 | Solyc11g068980 | 207 | 173 | 169 |
|  |  |  | AT1G18320 |  |  | 142 |  |
|  | Tim54 | YJL054W | NF | NF | 478 |  |  |
|  | Tim18 | YOR297C  YDR178W  YRL164W | NF | NF | 192  181  169 |  |  |
|  | Tim12 | YBR091C | NF | NF | 109 |  |  |
| PAM system | Pam16 | YJL104W | AT5G61880 | Solyc08g006120 | 149 | 113 | 114 |
|  |  |  | AT3G59280 | Solyc06g068820 |  | 116 | 114 |
|  |  |  |  | Solyc08g069210 |  |  | 221 |
|  |  |  |  | Solyc00g074210 |  |  | 149 |
|  | Pam18 | YLR008C | AT2G35795 | Solyc10g086040 | 168 | 112 | 112 |
|  |  |  | AT3G09700 | Solyc09g007390 |  | 112 | 112 |
|  |  |  | AT5G03030 | Solyc06g072330 |  | 112 | 110 |
|  | Mdj2** | YNL328C |  |  | 146 |  |  |
|  | Tim44 | YIL022W | AT2G20510^#^ | Solyc01g098230^#^ | 431 | 472 | 498 |
|  |  |  | AT2G36070 |  |  | 469 |  |
|  | Pam17 | YKR065C | NF | NF | 197 |  |  |
| SAM complex | Sam50/  Tob55 | YNL026W | AT3G11070 | Solyc04g079270 | 484 | 520 | 524 |
|  |  |  | AT5G05520 |  |  | 524 |  |
|  | Metaxin | NF | AT2G19080* | Solyc07g009320 |  | 315 | 327 |
|  | Sam35/  Tob38 | YHR083W | NF | NF | 329 |  |  |
|  | Sam37 | YMR060C | NF | NF | 327 |  |  |
|  | Mdm10 | YAL010C | NF | NF | 493 |  |  |
|  | Mim1 | YOL026C | NF | NF | 113 |  |  |
| MIA | Mia40 | YKL195W | AT5G23395 | Solyc04g008770 | 403 | 162 | 154 |
|  | Erv1 | YGR029W | AT1G49880 | Solyc11g071960 | 189 | 191 | 200 |
|  | Hot13 | YKL084W | NF | NF | 116 |  |  |
| OXA | Oxa1 | YER154W | AT5G62050 | Solyc08g008320 | 402 | 429 | 356 |
|  |  |  | AT2G46470 | Solyc08g082290 |  | 431 | 201 |
| Given is the central complex name (column 1), the name of the component (column 2), the accession number for the yeast (column 3), *A. thaliana* (column 4) and tomato (column 5) gene coding for the component and the amino acid length of the yeast (column 6) *A. thaliana* (column 7) and tomato protein (column 8). NF … no factor detected with the settings described in materials and methods. * [21]; ** same orthology group as Pam18, ^+^ depicts the correlation via syntenic analysis, ^#^ signifies correlation on the basis of expression pattern. | | | | | | | |
